# Cr(i)Cl as well as Cr^+^ are stabilised between two cyclic alkyl amino carbenes†
†Electronic supplementary information (ESI) available: Synthetic procedures, details of crystal structure refinements, magnetic measurements, EPR and theoretical investigations. CCDC 1034607, 1034608 and 1034606. For ESI and crystallographic data in CIF or other electronic format see DOI: 10.1039/c5sc00646e



**DOI:** 10.1039/c5sc00646e

**Published:** 2015-03-20

**Authors:** Prinson P. Samuel, Roman Neufeld, Kartik Chandra Mondal, Herbert W. Roesky, Regine Herbst-Irmer, Dietmar Stalke, Serhiy Demeshko, Franc Meyer, Vallyanga Chalil Rojisha, Susmita De, Pattiyil Parameswaran, A. Claudia Stückl, Wolfgang Kaim, Jonathan H. Christian, Jasleen K. Bindra, Naresh S. Dalal

**Affiliations:** a Institut für Anorganische Chemie , Georg-August-Universität , Tammannstrasse 4 , D-37077 , Göttingen , Germany . Email: hroesky@gwdg.de ; Email: dstalke@chemie.uni-goettingen.de ; Email: franc.meyer@chemie.uni-goettingen.de ; Fax: +49-551-39-33373, +49-551-39-33063 ; Tel: +49-551-39-33001, +49-551-39-33000, +49-551-39-33012; b Department of Chemistry , National Institute of Technology Calicut , 673601 , Kerala , India . Email: param@nitc.ac.in ; Tel: +91-495-228-5304; c Institut für Anorganische Chemie , Universität Stuttgart , Pfaffenwaldring 55 , D-70569 , Stuttgart , Germany; d Departments of Chemistry and Biochemistry , Florida State University , Tallahassee , FL 32306-4930 , USA . Email: dalal@chem.fsu.edu ; Fax: +1-850-644-8281 ; Tel: +1-850-644-3398; e National High Magnetic Field Laboratory , Florida State University , Tallahassee , FL 32306 , USA

## Abstract

Cr(i)Cl is a very unstable species. The present work describes the stabilisation of Cr(i)Cl in the low coordinate environment of cyclic alkyl(amino) carbene ligands and its synthetic application to yield an unprecedented cationic complex with a two coordinate Cr(i). One electron reduction of (cAAC)_2_CrCl_2_ (**1**) with equivalent amount of KC_8_ results in the formation of (cAAC)_2_CrCl (**2**), with a distorted trigonal planar configuration at the metal centre. SQUID, EPR and theoretical studies reveal a Cr(i) centre with *S* = 5/2 spin ground state for **2**. It represents the first example of a mononuclear Cr complex showing slow relaxation of magnetisation under an applied magnetic field. The chlorine atom in **2** is expected to be prone to further reactions with appropriate reagents. This qualifies **2** as a promising precursor for the preparation of various interesting complexes with Cr(i) in a low coordinate environment. The first example of this metathesis reaction is observed when **2** is treated with Na[B(C_6_H_3_(CF_3_)_2_)_4_] resulting in [(cAAC)_2_Cr]^+^[B(C_6_H_3_(CF_3_)_2_)_4_]^–^, a linear cationic complex with two coordinate Cr(i) and an *S* = 5/2 spin ground state.

## Abstract 2

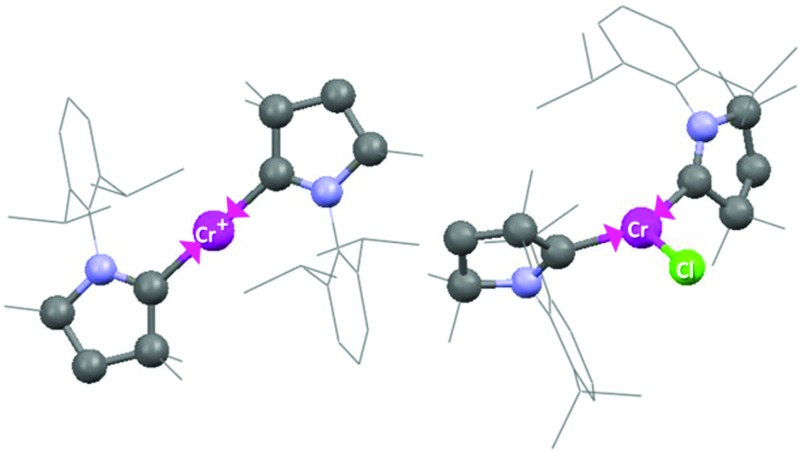
Complexes with two and three coordinate chromium(i).

## Introduction

Chromium is an important first-row transition element because of the broad utility of its compounds in catalysis and numerous other applications.^1^ In addition, the magnetic properties of chromium (Cr) are unique in the sense that it is the only element which shows antiferromagnetic ordering at temperatures below 38 °C while above this temperature it exhibits paramagnetism.^2^ A recent study revealed that elemental Cr exhibits special electrical properties similar to a magnet which would enable the application of antiferromagnets in spintronics.^3^ High coordinate, especially octahedral complexes dominate the coordination chemistry of Cr, but recent years have witnessed an interest in complexes with low coordinate chromium as well.^4^ One of the main strategies to obtain complexes with low-valent low coordinate chromium is to use sterically demanding ligands, which restrict metal atoms from achieving high-coordination polyhedra. Power and coworkers used a bulky monoanionic terphenyl ligand to synthesise ArCrCrAr (Ar = C_6_H_3_-2,6(C_6_H_3_-2,6-iPr_2_)_2_), the first stable binuclear compound with five-fold bonding between two Cr atoms.^5*a*^ However, a number of compounds with quintuply bonded Cr(i) centres have been synthesised since then using various ligands.^5b–g^ In a different strategy, other binuclear Cr(i) complexes of composition [nacnacCr]_2_(μ-X) (X = N_2_, C_6_H_6_; nacnac = CH(CMeNAr)_2_, Ar = C_6_H_3_-2,6-iPr_2_) were synthesised by Theopold and coworkers using a monoanionic chelating β-diketiminate ligand.^6^ Each Cr atom in these compounds is chelated by a terminal bidentate β-diketiminato and the two metal ions are bridged by a central N_2_ or benzene moiety. However, mononuclear β-diketiminato Cr(i) complexes were obtained by coordinating various acetylene derivatives to the Cr centre.^7^ Utilising an even more crowded terphenyl ligand Ar′ (Ar′ = C_6_H_1_-2,6-(C_6_H_2_-2,4,6-iPr_3_)_2_-3,5-iPr_2_) led to the formation of mononuclear compounds with two coordinate Cr(i) of compositions Ar′Cr(THF) and Ar′Cr(PPh_3_).^8^

In all of the above compounds the Cr(i) atoms are bound to a monoanionic and relatively bulky ligand. This consequently leaves the Cr(i) atom without a functional group, which could be further transformed to other important functionalities by appropriate metathesis reactions. In this respect our aim was to stabilise Cr(i)Cl species which, unlike Cr(ii) or Cr(iii) halides, do not exist under normal conditions.^9^ Various N-heterocyclic carbenes (NHC) have been reported to form adducts with Cr(ii)Cl_2_ and Cr(iii)Cl_3_, but they are not known to form stable adducts with Cr(i)Cl.^10^ At this juncture, it is worth noting that NHCs have their carbene carbon atom bonded to two N atoms which are σ-withdrawing and π-donating whereas in cyclic alkyl amino carbenes (cAACs) one of the nitrogen atoms is replaced by a σ-donating quarternary carbon atom. This allows cAACs to exhibit more nucleophilic and electrophilic character than NHCs and hence utilising cAACs will be advantageous to stabilise unstable species with low-valent metal centres.^11^ In this direction, we have prepared (cAAC)_2_CrCl, a compound in which Cr(i)Cl is stabilised by two flanking cAACs. This compound features the first stable Cr(i)Cl entity having Cr(i) in a three coordinate non-chelating ligand environment. Such a compound is anticipated to offer enormous scope for the preparation of various other Cr(i) derivatives by replacing the chlorine atom with suitable functional groups of interest. Consequently an attempt to substitute the chlorine atom with an anionic group of extremely high steric hindrance may result in the stabilisation of a Cr^+^ species in the coordination environment of two neutral cAACs. A recent report showed an anionic complex with Cr(i) in the two coordinate environment of monoanionic amido ligand, N[Si(iPr)_3_]Dipp (Dipp = C_6_H_3_-2,6-iPr_2_).^12^ However, Cr(i) cationic complexes so far known are stabilised only in high coordinate environment. The best known of this kind are π-arene complexes of Cr(i) as well as chromium carbonyl complexes with phosphorus donor ligands.^13^ These Cr(i) cationic complexes have been found to be effective in catalytic ethylene tetramerisation and trimerisation reactions. However, there is no report of a cationic compound with a two coordinate Cr(i) ion and so we became interested in synthesising such a novel compound. Reaction of (cAAC)_2_CrCl with Na[B(C_6_H_3_(CF_3_)_2_)_4_] resulted in the formation of [(cAAC)_2_Cr]^+^[B(C_6_H_3_(CF_3_)_2_)_4_]^–^, a cationic two coordinate Cr(i) complex. It should also be noted that low coordinate transition metal complexes with high symmetry and low metal oxidation state attract a lot of interest because of their potentially interesting magnetic properties, including large magnetic relaxation barriers. This is because the remaining near-degeneracy of d-orbitals in such low coordinate systems, in combination with a proper number of d-electrons, can give rise to significant unquenched orbital angular momentum.^14^ Hence the magnetic properties of some of the new Cr complexes have been investigated.

## Results and discussion

**Scheme 1:**
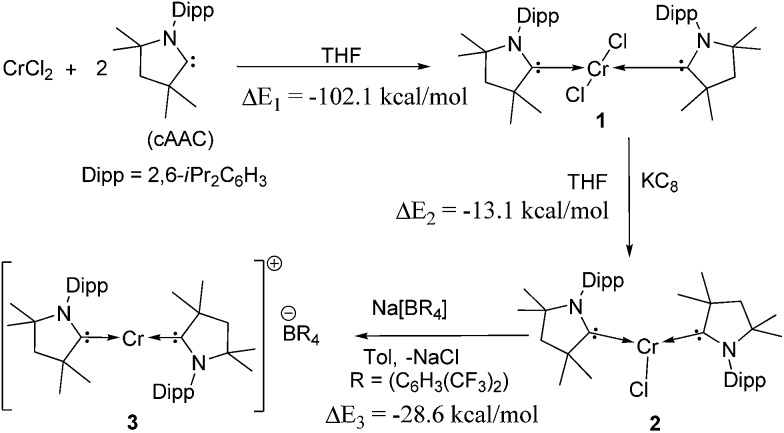
Synthesis of compounds **1–3**. Gas phase reaction energies, Δ*E* in kcal mol^–1^ are calculated at the M06/def2-TZVPP//BP86/def2-SVP level of theory.^15^ Δ*E*_2_ is the reaction energy of **1** with K, considering KC_8_ as a source of the latter.

**Fig. 1:**
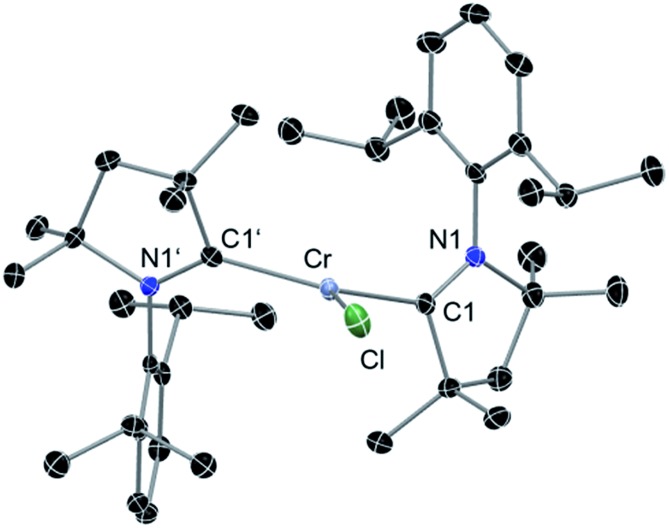
Molecular structure of **2**. Hydrogen atoms are omitted for clarity. Anisotropic displacement parameters are depicted at the 50% probability level. Selected bond lengths [Å] and angles [°]. Calculated values at the BP86/def2-SVP level of theory are given in square brackets.^15^ Cr–Cl, 2.366(1) [2.332]; Cr–C1, 2.084(2) [2.091]; Cr–C1′, 2.093(2) [2.091]; C1–N1, 1.333(2) [1.350]; C1′–N1′, 1.333(2) [1.350]; C1–Cr–Cl, 112.70(5) [106.3]; C1′–Cr–Cl, 110.11(5) [106.3]; C1–Cr1–C1′, 137.17(6) [147.3].

Reaction of two equivalents of cAAC with CrCl_2_ in THF results in the formation of (cAAC)_2_CrCl_2_ (**1**), a pink compound in high yield (Scheme 1). The high exothermicity (–102.1 kcal mol^–1^) for the formation of **1** from CrCl_2_ and cAAC supports the experiment. The molecular structure of **1** shows Cr–C (2.180 Å) and Cr–Cl (2.339 Å) bond distances comparable to the corresponding bond lengths in (NHC)_2_CrCl_2_ reported earlier^10*a*^ (see ESI† for structural details of **1**). The reaction of equivalent amounts of **1** and KC_8_ in THF afforded (cAAC)_2_CrCl (**2**) as a green product. The abstraction of Cl from **1** by K is calculated to be exothermic by –13.1 kcal mol^–1^. **2** crystallises in the triclinic space group *P*1. The Cr atom in **2** features a distorted trigonal planar coordination geometry. The molecular structure of **2** is shown in Fig. 1. The distance of the chromium atom from the C1–Cl–C1′ plane is 0.018 Å, which is very close to the ideal planar geometry around the Cr atom. The Cr–C bond lengths in **2** are 2.084(2) and 2.093(2) Å which are shorter than the corresponding distances in **1**. However, the Cr–Cl bond length is 2.366(1) Å which is close to the corresponding value for **1**. Unlike in the case of **1**, the cAAC ligands in **2** are arranged *trans* with respect to the position of the N atoms.

**Fig. 2:**
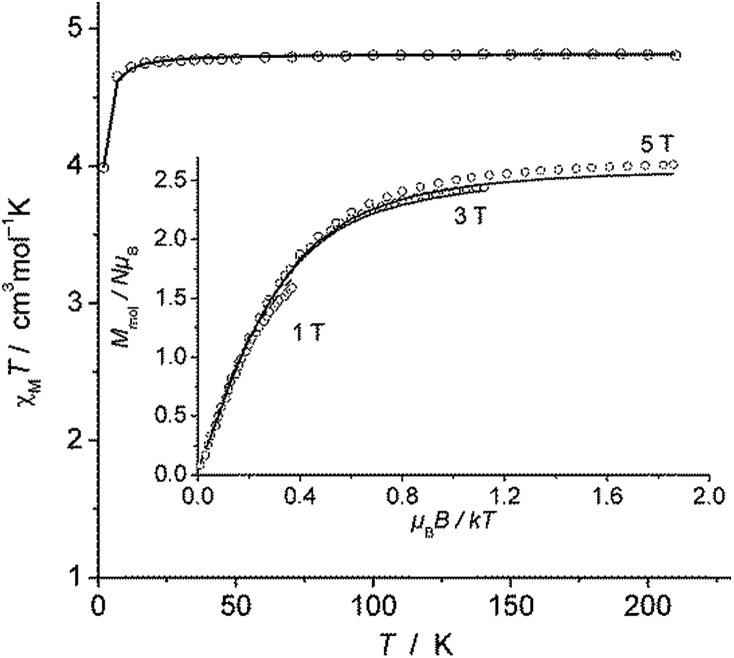
*χ*_M_ *T versus T* plot for **2**. Inset: VTVH magnetisation measurements as *M*_mol_ *versus μ*_B_ *B*/*kT*. Solid lines represent the calculated curve fits (see text).

The magnetic susceptibility measurement of **2** (Fig. 2) shows a *χ*_M_ *T* value of 4.81 cm^3^ mol^–1^ K at 210 K, which is slightly higher than the expected spin-only value for a *S* = 5/2 system (4.375 cm^3^ mol^–1^ K). *χ*_M_ *T* remains nearly constant down to 20 K but drops to 4.00 cm^3^ mol^–1^ K at 2 K, which is likely due to zero field splitting (*vide infra*). No temperature dependence of *χ*_M_ *T* was observed between 20 K and 210 K, indicating that only the *S* = 5/2 spin state is populated in this temperature range. The experimental *χ*_M_ *T vs. T* as well as the variable temperature-variable field (VTVH) magnetisation data were simultaneously modelled using the anisotropic spin Hamiltonian with Zeeman splitting as well as axial (*D*) and rhombic (*E*) zero-field splitting as given in eqn (1).^16^
1
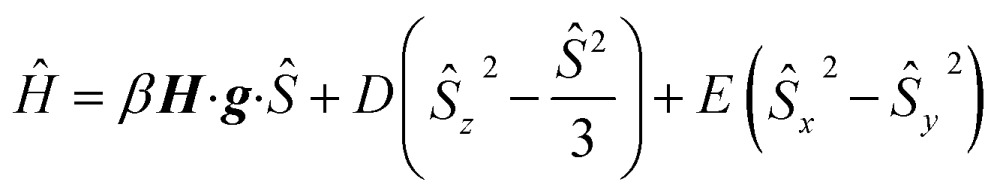



In this Hamiltonian, ***H*** is the magnetic field vector, ***g*** is the Zeeman tensor, and the other terms pertain their usual meaning.^17^ The best fit values are *g*_*x*_ = *g*_*y*_ = 1.93, *g*_*z*_ = 2.41 and *D* = 0.5 cm^–1^. The rhombic ZFS parameter *E* was fixed to zero to avoid any overparametrisation. It was also possible to simulate the experimental data using a negative *D* with *g*_*x*_ = *g*_*y*_ > *g*_*z*_. Thus, the SQUID measurements were inconclusive with respect to a proper assignment of the sign of *D* in this case, likely due to small magnitude of this parameter (∼1 cm^–1^).

**Fig. 3:**
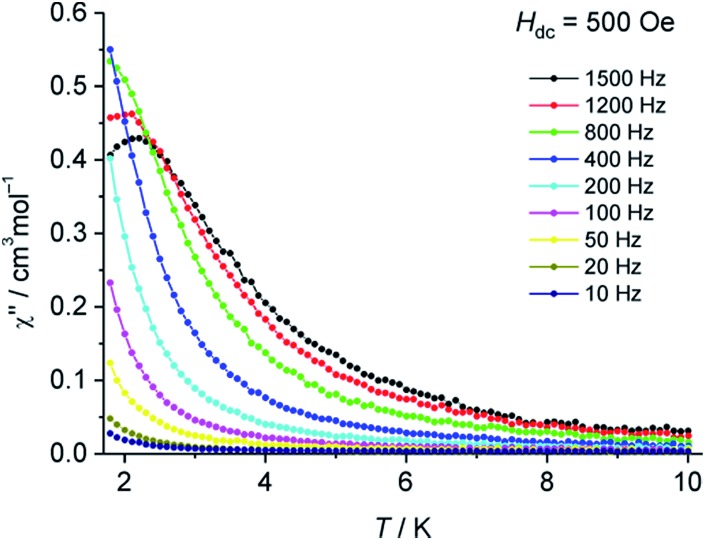
Temperature dependence of *χ*′′ for **2** at various frequencies with an applied dc field of *H*_dc_ = 500 Oe.

Alternating current (ac) magnetic susceptibility measurements at various frequencies were performed, both in the absence of a direct current (dc) magnetic field as well as with applied dc fields (*H*_dc_ = 250–3000 Oe). Application of dc fields revealed a frequency dependence in the imaginary part of the magnetic susceptibility (*χ*′′) (Fig. 3 and S6†), thus indicating slow relaxation of magnetisation in **2** at low temperatures. Although 3d transition metal based mononuclear single molecule magnets (SMMs) have been known since 2010,^18^ so far, only mononuclear SMMs containing Fe(i, ii, iii), Co(ii), Ni(i) or Mn(iii) ions have been reported.^19^ Significant efforts have been made to understand the magnetic behavior of homometallic and heterometallic clusters of chromium in the recent past.^20^ To the best of our knowledge, this is the first report on slow relaxation of magnetisation for a mononuclear Cr complex suggesting SMM behavior.

**Fig. 4:**
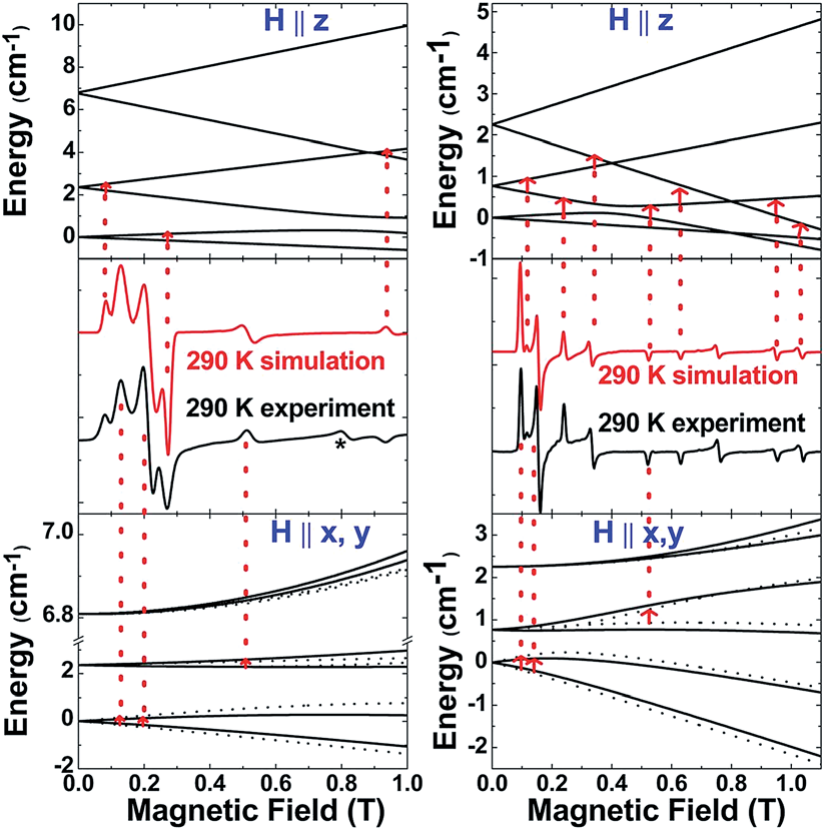
Experimental and simulated spectra of **2** (left) and **3** (right) at 290 K. Also shown are the energy-level diagrams with the magnetic field oriented parallel (*H*∥*z*) and perpendicular (*H*∥*x*,*y*) to the principal symmetry axis of the molecule. The red arrows mark the EPR transition assignments. For **2** the * indicates a peak tentatively attributed to level-crossing effects.

The *S* = 5/2 spin state of (cAAC)_2_CrCl derived from SQUID measurements is further supported by X-band (9.45 GHz) EPR measurements on a finely ground polycrystalline powder of **2**. The spectra were analysed using a locally developed computer programme,^21^ which extracts the magnetic parameters by diagonalising the Hamiltonian matrix shown in eqn (1). Fig. 4 (left) displays the room-temperature experimental spectrum (middle segment, black trace) of **2**. The simulated spectrum (middle segment, red trace) was obtained using *S* = 5/2, *g*_*x*_ = 1.47, *g*_*y*_ = 1.40, *g*_*z*_ = 2.70, |*D*| = 1.12 cm^–1^ and *E*/*D* = 0.07. The top and bottom portions show the energy level diagrams with the magnetic field oriented along the principal symmetry axis of the molecule (*H*∥*z*) and along the perpendicular (*H*∥*x*,*y*) directions. The red arrows indicate the EPR transition assignment. The agreement between the observed and simulated spectrum is quite satisfactory, although the experimental spectrum shows a peak at 0.8 T, which could not be simulated, but could be tentatively ascribed to level-crossing effects based on an excellent agreement of the data for **3** (*vide infra*). Nonetheless, the simulation parameters are in agreement with the magnetic susceptibility data and provide further validity to the assignment of a Cr(i) metal centre with *S* = 5/2 and small zero-field splitting energy.

**Fig. 5:**
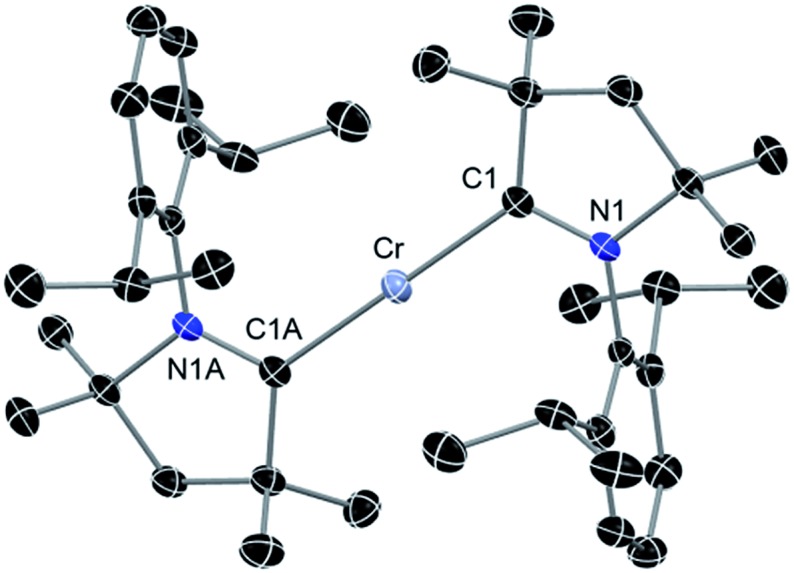
Molecular structure of cationic part of **3**. Hydrogen atoms are omitted for clarity. Anisotropic displacement parameters are depicted at the 50% probability level. Primes (′) represent the atoms of the second molecule present in the asymmetric unit. Selected bond lengths [Å] and angles [°]. Calculated values at the BP86/def2-SVP level of theory are given in square brackets.^15^ Cr–C1, 2.134(2) [2.152]; Cr′–C1′, 2.136(2) [2.152]; C1–N1, 1.303(2) [1.324]; C1′–N1′, 1.305(2) [1.324]; C1–Cr–C1A, 180.0 [180.0].

The above discussion indicates that in **2** the Cr(i)Cl species which would otherwise be very unstable is stabilised by the donor electrons of the cAACs. This is further supported by the complementary computations (see below). Replacement of chlorine atom in **2** with other functional groups is an open challenge which may lead to a bouquet of new complexes with low coordinate Cr(i) with potential applications. In the first reactivity study of this series, we have found that the reaction of **2** with equivalent amounts of Na[B(C_6_H_3_(CF_3_)_2_)_4_] results in the elimination of NaCl and thereby the formation of the ionic compound [(cAAC)_2_Cr]^+^[B(C_6_H_3_(CF_3_)_2_)_4_]^–^ (**3**) as a pale green solid. The formation of **3** *via* the abstraction of Cl^–^ by Na^+^ is exothermic by –28.6 kcal mol^–1^. Single crystals of **3** suitable for X-ray diffraction were obtained from a saturated solution in toluene at –35 °C. **3** crystallises in the triclinic space group *P*1. The molecular structure of the cation in **3** is given in Fig. 5 (see ESI† for full molecular structure). The geometry around Cr is strictly linear and the Cr atom is located on a crystallographic inversion centre. However, there are two crystallographically different molecules with Cr–C bond distances of 2.134(2) and 2.136(2) Å which are a little longer than the corresponding bond distances of **2**. The EPR spectrum of **3** was measured and simulated similarly to **2** which resulted in an excellent fit using the parameters: *S* = 5/2, *g*_isotropic_ = 2.00, |*D*| = 0.37 cm^–1^ and *E*/*D* = 0.06, as shown in Fig. 4 (right).

Quantum mechanical calculations at the M06/def2-TZVPP//BP86/def2-SVP level of theory^15^ show that the quintet state of complex **1** is lower in energy than the triplet and singlet electronic states by 59.2 and 63.6 kcal mol^–1^, respectively. The sextet state of **2** is more stable by 5.8 and 38.5 kcal mol^–1^ whereas the sextet state of **3** is more stable by 33.3 and 66.6 kcal mol^–1^ as compared to their quartet and doublet states, respectively. The calculated geometrical parameters of **1**, **2** and **3** in their respective high-spin states are also closest to those of the crystal structures (Fig. S12–S14 and Tables S3–S5†). The C–N bond lengths and Wiberg bond indices in the free cAAC ligand (1.320 Å, 1.50), complexes **1** (1.330 Å, 1.47), **2** (1.350 Å, 1.37) and **3** (1.324 Å, 1.50) suggest that the Cr → π*cAAC back donation is less in these complexes (Table S6–S8†), while in **2** a relatively higher back donation is observed. The geometrical analysis is in line with the following bonding description based on molecular orbital and NBO analyses.^15^

The valence electron (VE) count of Cr(ii) in **1**, Cr(i) in **2** and Cr(i) in **3** are 12, 11 and 9 respectively. Apart from the Cr–ligand bond formation in these high spin complexes, four, five and five valence electrons occupy Cr-based d-orbitals in **1**, **2** and **3** respectively (Fig. S9–S11†). These singly occupied d-orbitals are responsible for Cr → π*cAAC back donation. Hence, the extent of Cr → π*cAAC back donation can be understood from NBO spin density. The calculated NBO spin densities on the Cr atoms in complexes **1**, **2** and **3** are 3.85, 4.25 and 4.65 respectively (Table S9†). The relatively less spin density on the Cr atom in **2** as compared to that of **3** can be attributed to relatively higher Cr → π*cAAC back donation in **2**, which is also shown in its SOMO+3 and SOMO+4 (Fig. S10†). However, only one MO shows Cr → π*cAAC back donation in **3** (SOMO+2 in Fig. S11†) and in **1** (SOMO+3 in Fig. S9†). The lowest positive group charge of cAAC in **2** (0.01*e*) as compared to **1** (0.23*e*) and **3** (0.23*e*) also corroborates well with the MO analysis.

The dissociation energies of one Cr–C_cAAC_ bond in **1**, **2** and **3** are 51.1, 48.6 and 79.7 kcal mol^–1^, respectively. The highest Cr–C_cAAC_ bond strength in **3** can be qualitatively attributed to the higher charge of the complex, while the weaker Cr–C_cAAC_ bond strength in **2** as compared to **1** can be attributed to the lower oxidation state of Cr in **2**. Moreover, the SOMOs showing the σ-antibonding interaction between Cr and Cl^–^ in **2** indicate easy removal of Cl^–^ ligand for the formation of complex **3**. The dissociation energies of a Au–C_cAAC_ bond in Au(cAAC)_2_, Cu–cAAC bond in Cu(cAAC)_2_ and Co–C_cAAC_ in Co(cAAC)_2_ are 45.6, 48.7 and 64.3 kcal mol^–1^, respectively.^22^ These molecules show significant amount of π-back donation, a situation very different from the present Cr complexes. Note that the NBO data such as C–N bond order, partial charges and spin density give only the relative strength of donor–acceptor interactions in these complexes. It does not necessarily correlate with the dissociation energy of the Cr–C_cAAC_ bond, which depends on the other energy components as well.^22*d*^ Thus, **1**, **2** and **3** are examples of complexes with low coordinate and low-valent Cr in their highest spin states mainly stabilised by the σ-donation of the cAAC ligands.

In order to understand the extent of Cr → π*cAAC back donation as a function of spin states, we have calculated the C–N bond order and cAAC group charges of complex **3** in the sextet, quartet and doublet spin states (Table S8†). The C–N bond order in the sextet state (1.50) is the same as that of free cAAC, whereas the corresponding bond orders in quartet and doublet states are 1.34 and 1.38, respectively. If the C–N bond order can be considered as the measure of Cr → π*cAAC back donation, the relative back donation is in the order: quartet > doublet > sextet. This is in line with the calculated cAAC group charges *viz.*, 0.23 for sextet, 0.05 for quartet and 0.12 for the doublet. Hence, rather than the Cr → π*cAAC back donation, the sextet spin state plays a more significant role for the stability of the low coordinate chromium of **3**. Similar analysis in complexes **1** and **2** also support the same conclusion (Tables S6 and S7†).

## Conclusions

This work presents two novel low coordinate Cr(i) complexes **2** and **3**. The X-ray crystal structure, magnetic, EPR and theoretical studies show a rare Cr(i)Cl species that is stabilised by the coordination of two cAAC ligands. The presence of a chlorine atom in the three coordinate Cr(i) complex **2** is expected to make it a good precursor for the preparation of various Cr(i) compounds of potential interest. Attempts are in progress in our laboratory to replace the chlorine atom with other important elements such as fluorine and hydrogen. **2** represents the first example of a mononuclear Cr complex showing slow relaxation of magnetisation under an applied magnetic field. This work also shows the utility of **2** as a precursor for preparing **3**, a hitherto elusive Cr(i) cationic compound in a two coordinate neutral ligand environment. The magnetic parameters for **3** are surprisingly quite different from those of **2**, while the total spin stays the same, *S* = 5/2, thus exhibiting the possible control over the magnetism that can be synthetically achieved in the compounds. However, potential applications of the coordinately unsaturated Cr(i) sites in **2** and **3** are still to be investigated.
