# Supplementary material for: Cr(i)Cl as well as Cr+ are stabilised between two cyclic alkyl amino carbenes
Source: Chem Sci. 2015 Mar 20;6(5):3148–53. doi: 10.1039/c5sc00646e (PMC5490427; doi:10.1039/c5sc00646e)
Supplement: Supplementary file 1 [file SC-006-C5SC00646E-s001.pdf]

Electronic Supplementary Material (ESI) for Chemical Science.
This journal is © The Royal Society of Chemistry 2015

Supporting Information

Cr(I)Cl as well as Cr^+ are stabilised between two cyclic alkyl amino carbenes

Prinson P. Samuel,[1] Roman Neufeld,[1] Kartik Chandra Mondal,[1] Hebert W. Roesky,[1]* Regine Herbst-Irmer,[1] Dietmar Stalke,[1]* Serhiy Demeshko,[1] Franc Meyer,[1]* Vallyanga Chalil Rojisha,[2] Susmita De,[2] Pattiyil Parameswaran,[2]* A. Claudia Stückl,[1] Wolfgang Kaim,[3] Jonathan H. Christian,[4] Jasleen K. Bindra[4] and Naresh S. Dalal [4,5]*

[1]Institut für Anorganische Chemie, Georg-August-Universität, Tammannstrasse 4, D-37077, Göttingen, Germany.

[2]Department of Chemistry, National Institute of Technology Calicut, 673601, Kerala, India.

[3]Institut für Anorganische Chemie, Universität Stuttgart, Pfaffenwaldring 55, D-70569, Stuttgart, Germany.

[4]Departments of Chemistry and Biochemistry, Florida State University, Tallahassee, FL 32306, USA.

[5] National High Magnetic Field Laboratory, Florida State University, Tallahassee, FL 32306, USA.

Contents

S1. Syntheses of **1**-**3**

S2. Crystallographic information

S3. Magnetic measurements

S4. EPR measurements

S5. Theoretical calculations

S1. Syntheses of 1-3

Syntheses were carried out under an inert gas atmosphere of argon in oven-dried glassware using standard Schlenk techniques. Other manipulations were accomplished in a dinitrogen filled glove box. Solvents were purified by MBRAUN solvent purification system MB SPS-800. All chemicals were purchased from Aldrich and used without further purification. cAAC was prepared following the reported procedure.[1] Elemental analyses were carried out in the Analytisches Labor der Anorganischen Chemie der Universität Göttingen.

Synthesis of **1**: To $CrCl_2$ (0.246 g, 2 mmol) and cAAC (1.14 g, 4 mmol), THF (8 mL) was added at room temperature and the mixture was stirred overnight. The turbid solution was filtered though a frit and **1** was collected as a pink precipitate. Storing a saturated THF solution of **1** at -4 ^{o}C afforded crystals suitable for X-ray diffraction. (Yield: 88 %, 1.22 g). Mp 156 ^{o}C (decomp.) Elemental analysis (%) calcd for $C_{40}H_{62}Cl_2CrN_2$ (692.37): C, 69.24, H, 9.01, N, 4.04 Found: C, 68.88, H, 9.21, N, 4.09.

Synthesis of **2**: Precooled THF (30 mL, -78 ^{o}C) was added to **1** (0.693 g, 1 mmol) and KC_8 (0.135 g, 1 mmol) and stirred for 2 h while allowing the temperature to rise slowly. After reaching room temperature, stirring was continued for another 45 min and the solution was filtered to remove the graphite. The green solution was subjected to reduced pressure to remove THF and the dark green residue was extracted with toluene (50 mL). The solution was concentrated and stored at -32 ^{o}C to afford single crystal of **2** suitable for X-ray diffraction. (Yield: 69 %, 0.45 g). Mp 127 ^{o}C (decomp.) Elemental analysis (%) calcd for $C_{40}H_{62}ClCrN_2$ (657.40): C, 72.97, H, 9.49, N, 4.25 Found: C, 72.79, H, 9.85, N, 4.16.

Synthesis of **3**: Precooled toluene (30 mL, -78 ^{o}C) was added to **2** (0.200 g, 0.30 mmol) and $Na[B(C_6H_3(CF_3)_2)_4]$ (0.268 g, 0.30 mmol) while stirring. The temperature of the solution was allowed to rise slowly within 1.5 h to room temperature. After reaching room temperature the solution was allowed to stir for additional 30 min. The solution was again cooled to 0 ^{o}C and stirred for 15 min. The precipitated pale green compound **3** was collected by filtration and single crystals suitable for X-ray diffraction were obtained from a saturated solution in toluene at -32 ^{o}C. (Yield: 85 %, 0.38 g). Mp 124 ^{o}C (decomp.) Elemental analysis (%) calcd for $C_{72}H_{74}BCrF_{24}N_2$ (1485.50): C, 58.19, H, 5.02, N, 1.88 Found: C, 57.71, H, 5.39, N, 1.62.

S2. Crystallographic information

Suitable single crystals were selected from the mother liquor in the Schlenk flask and covered with perfluorinated polyether oil on a microscope slide, which was cooled with a nitrogen gas flow using the X-Temp2 device.[2] The diffraction data of 1, 2 and 3 were collected at 100 K on a Bruker D8 three circle diffractometer equipped with a SMART APEX II CCD detector and a microfocus source[3] with INCOATEC Quazar mirror-monochromated Mo-Kα radiation (λ = 0.71073 Å). The data were integrated with SAINT[4] and a multi-scan absorption correction with SADABS[5] was applied. The structures were solved by direct methods (SHELXS-97)[6a] and refined against all data by full-matrix least-squares methods on F^2 (SHELXL2013)[6b,c] within the SHELXLE GUI.[6d] The hydrogen atoms were refined isotropically on calculated positions using a riding model with their U_{iso} values constrained to 1.5 U_{eq} of their pivot atoms for terminal sp^3 carbon atoms and 1.2 times for all other carbon atoms. All non-hydrogen-atoms were refined with anisotropic displacement parameters. Disordered moieties were refined using distance restraints and anisotropic displacement parameter restraints (SIMU, RIGU and SAME).[6c]

Table S1. Crystal and Structure Refinement parameters for compounds **1-3**.

Parameters	**1**	**2**·C_7H_8	**3**·1.5C_7H_8
CCDC-No.	1034607	1034608	1034606
Empirical formula	C_{40} H_{62} Cl_2 Cr N_2	C_{47} H_{70} Cl Cr N_2	$C_{82.50}$ H_{86} B Cr F_{24} N_2
Formula Weight	693.81	750.50	1624.34
Crystal system	Monoclinic	triclinic	triclinic
Space group	*C2/c*	*P -1*	*P -1*
Unit cell dimensions	a = 16.958(3) Å<br>b = 10.973(2) Å<br>c = 22.073(5) Å<br>α = 90 °<br>β = 108.91(2)°<br>γ = 90 °	a = 9.636(2) Å<br>b = 12.053(2) Å<br>c = 19.940(3) Å<br>α = 102.29(2)°<br>β = 95.37(2)°<br>γ = 106.71(2)°	a = 13.468(2) Å<br>b = 14.272(2) Å<br>c = 20.836(3) Å<br>α = 85.98(2)°<br>β = 89.96(2)°<br>γ = 87.39(2)°
Volume, Z	3885.7(14) Å^3, 4	2137.4(7) Å^3, 2	3991.0(10) Å^3, 2

Density (calcd)	1.186 Mg/m^3	1.166 Mg/m^3	1.352 Mg/m^3
Absorption coefficient	0.461 mm^{-1}	0.363 mm^{-1}	0.244 mm^{-1}
F (000)	1496	814	1680
Crystal size	0.20 x 0.14 x 0.10 mm^3	0.17 x 0.17 x 0.07 mm^3	0.10 x 0.10 x 0.05 mm^3
θ range for data collection	1.950 to 26.376°	1.060 to 26.362°.	1.432 to 25.350°.
Limiting indices	-21<=h<=21, -13<=k<=13, -27<=l<=27	-12<=h<=12, -15<=k<=15, -24<=l<=24	-16<=h<=16, -17<=k<=17, -25<=l<=25
Reflections collected	36281	71070	84083
Independent reflections	3982 [R(int) = 0.0309]	8692 [R(int) = 0.0523]	14604 [R(int) = 0.0526]
Completeness to θ	100 % (θ = 25.242°)	99.9 %(θ = 25.242°)	100 %(θ = 25.242°)
Refinement method	Full-matrix least-squares on F^2	Full - matrix least - squares on F^2	Full - matrix least - squares on F^2
Data/restraints/ parameters	3982 / 233 / 268	8692 / 0 / 477	14604 / 2848 / 1164
Goodness - of - fit on F^2	1.058	1.043	1.012
Final R indices [I>2σ (I)]	R_1 = 0.0277, wR_2 = 0.0697	R_1 = 0.0341, wR_2 = 0.0752	R_1 = 0.0380, wR_2 = 0.0802
R indices (all data)	R_1 = 0.0330, wR_2 = 0.0727	R_1 = 0.0467, wR_2 = 0.0814	R_1 = 0.0614, wR_2 = 0.0898
Largest diff. peak and hole	0.301 and -0.351 e.Å^{-3}	0.307 and -0.375 e.Å^{-3}	0.306 and -0.353 e.Å^{-3}

Molecular structure of 1

The molecular structure of **1** is shown in Figure S1. It crystallizes in space group C_2/c with half a molecule in the asymmetric unit the second half generated by a two-fold axis. The coordination geometry around chromium is distorted square planar and the two cAACs assume a *cis* arrangement with respect to the nitrogen atoms adjacent to the carbene carbon atoms. This cis arrangement is facilitated by the less hindering steric property of the cAAC ligands employed in this work. The C-Cr (2.180 Å) and Cr-Cl (2.339 Å) bond distances found in **1** is comparable to the corresponding bond lengths in $(NHC)_2CrCl_2$ compounds reported earlier.[7]

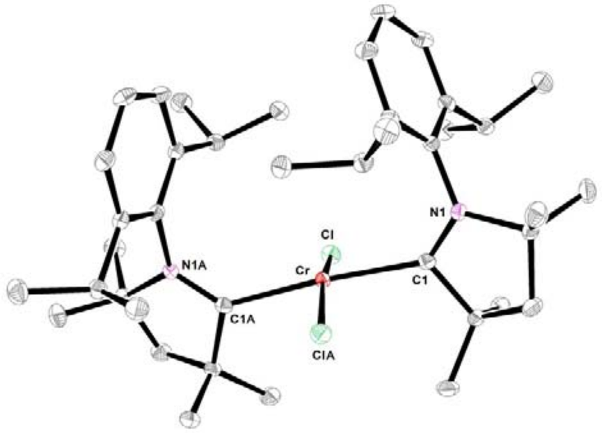

Figure S1. Molecular structure of **1**. Hydrogen atoms are omitted for clarity. Anisotropic displacement parameters are depicted at the 50% probability level. Selected bond lengths [Å] and angles [°]. Calculated values at the BP86/def2-SVP level of theory are given in square brackets. Cr–Cl, 2.339(1) [2.343, 2.345]; Cr–C1, 2.180(2) [2.146, 2.150]; C1–N1, 1.311(2) [1.331,1.330]; C1A–Cr–Cl, 84.72(4) [83.3], C1–Cr–Cl, 94.78(4) [96.0], C1–Cr–C1A, 175.61(7) [170.2].

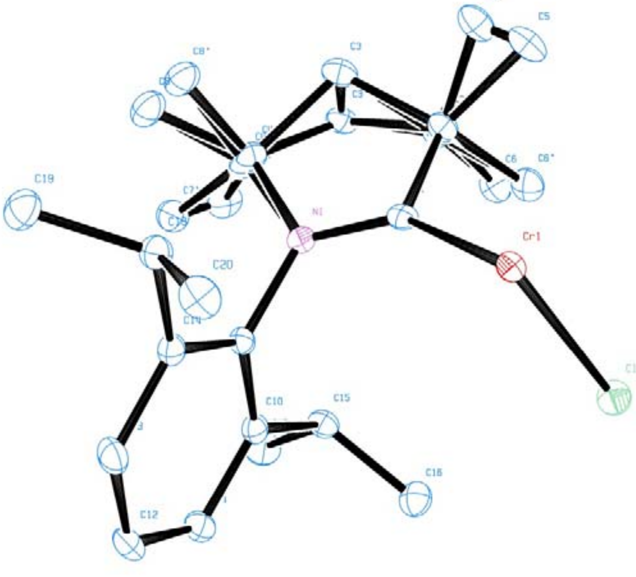

Figure S2. Assymmetric unit in the crystal structure of compound **1** with the monoclinic space group C_2/c. Assymmetric unit contains only half of the molecule and the other half is symmetry generated by –x, y, ½–z. Hydrogen atoms are omitted for clarity. Anisotropic displacement parameters are depicted at the 50% probability level.

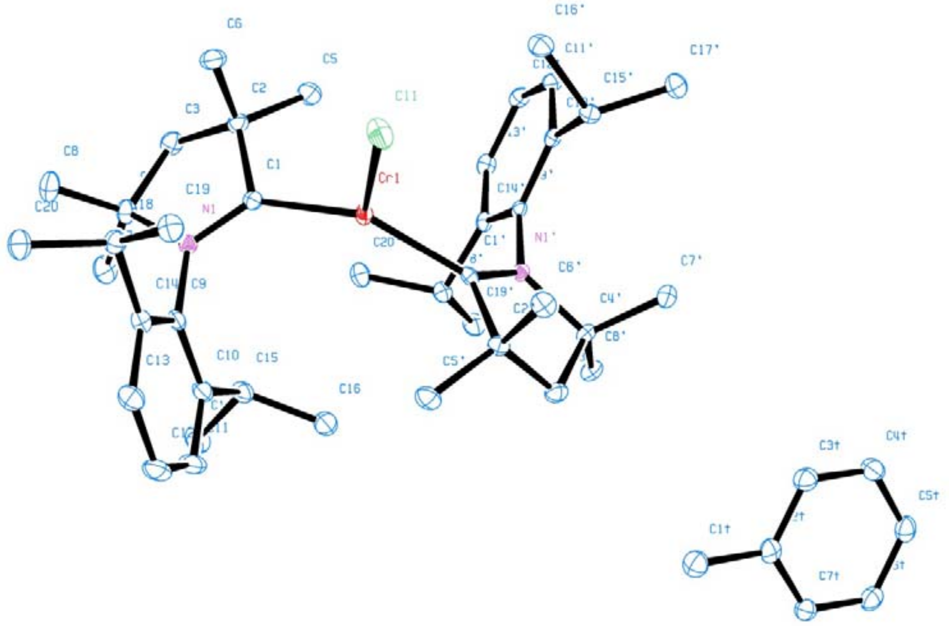

Figure S3. Assymmetric unit in the crystal structure of **2**·C_7H_8 with the triclinic space group *P*-1. Figure shows that the asymmetric unit contains one molecule of **2** and a toluene molecule. Hydrogen atoms are omitted for clarity. Anisotropic displacement parameters are depicted at the 50% probability level.

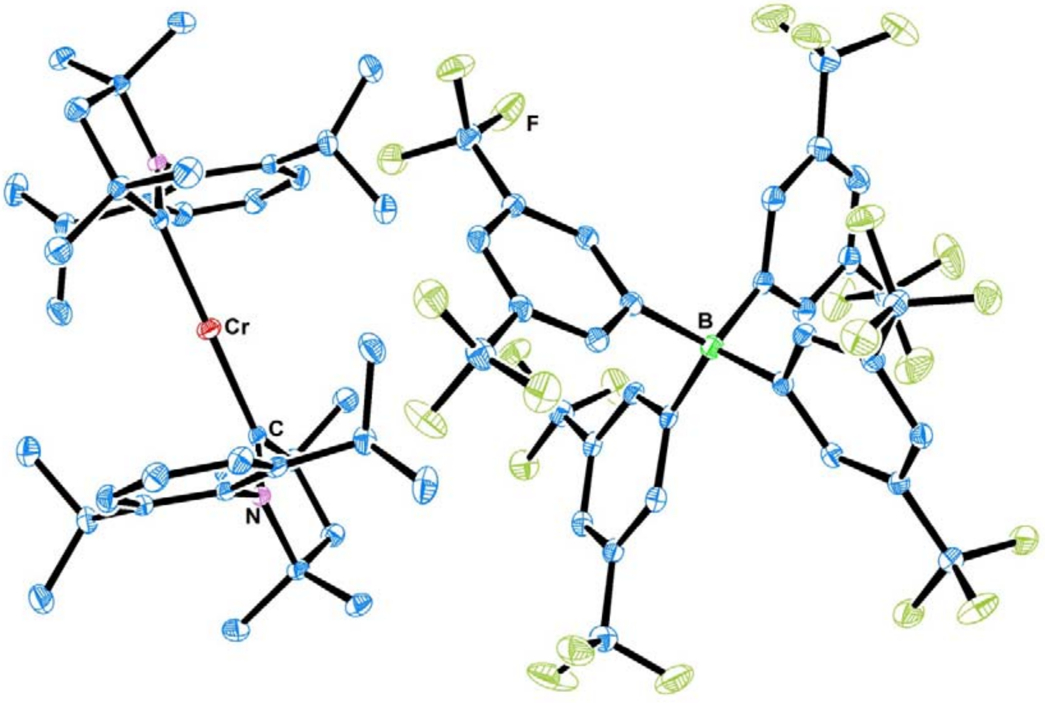

Figure S4. Complete molecular structure of **3**. Hydrogen atoms are omitted for clarity. Anisotropic displacement parameters are depicted at the 50% probability level.

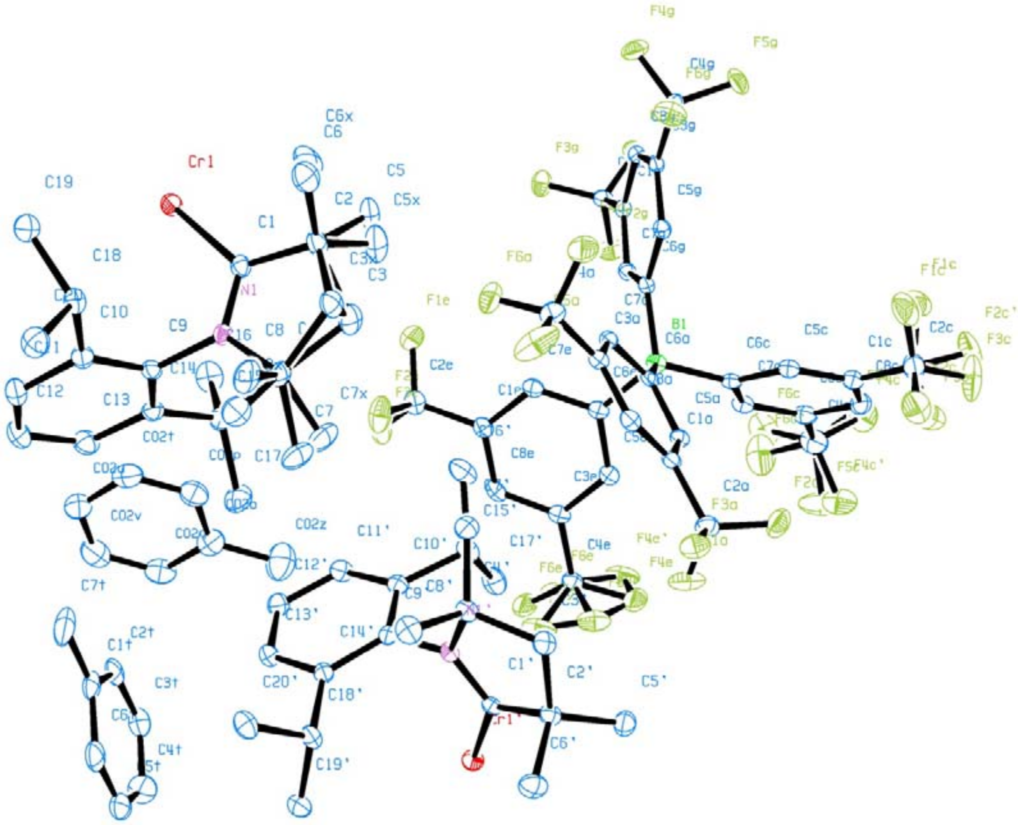

Figure S5. Assymmetric unit in the crystal structure of **3**·1.5C_7H_8 with the triclinic space group *P*-1. The asymmetric unit cell contains two half Cr containing catic part of **3**, one B containg anionic part of **3** and one and a half toluene molecules. Half of the Cr containg cations are symmetry generated by 1–x, 1–y, –z and 1–x, –y. 1–z. Hydrogen atoms are omitted for clarity. Anisotropic displacement parameters are depicted at the 50% probability level.

S3. Magnetic measurements

Temperature-dependent magnetic susceptibility measurements of **2** were carried out with a *Quantum-Design* MPMS-XL-5 SQUID magnetometer equipped with a 5 Tesla magnet in the range from 210 to 2 K in a magnetic field of 0.5 T. The polycrystalline sample was contained in a gel bucket, covered with a few drops of low viscosity perfluoropolyether based inert oil Fomblin YL VAC 25/6 to fix the crystals, and fixed in a non-magnetic sample holder. Each raw data file for the measured magnetic moment was corrected for the diamagnetic contribution of the gel bucket and of the inert oil. The molar susceptibility data were corrected for the diamagnetic contribution using the Pascal constants and the increment method according to Haberditzl.[8] Temperature-independent paramagnetism (*TIP*) was included according to $\chi_{calc} = \chi + TIP$. Before simulation, the experimental data were corrected for $TIP = 20 \cdot 10^{-6}$ cm^3mol^{-1}.

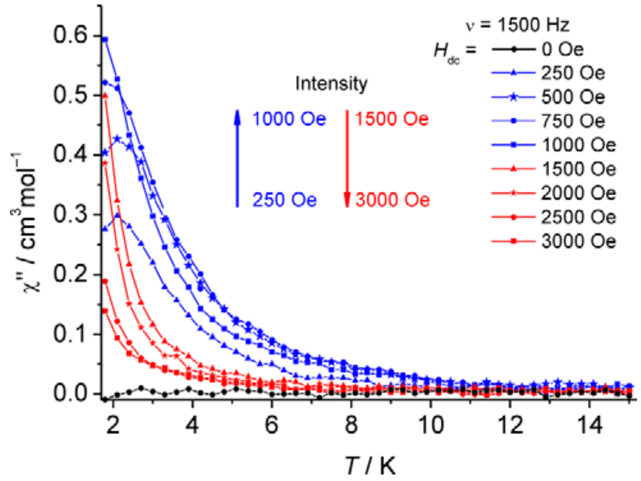

Figure S6. Temperature dependence of χ'' at 1500 Hz in the absence of a dc fields and with applied dc fields of H_{dc} = 250 - 3000 Oe.

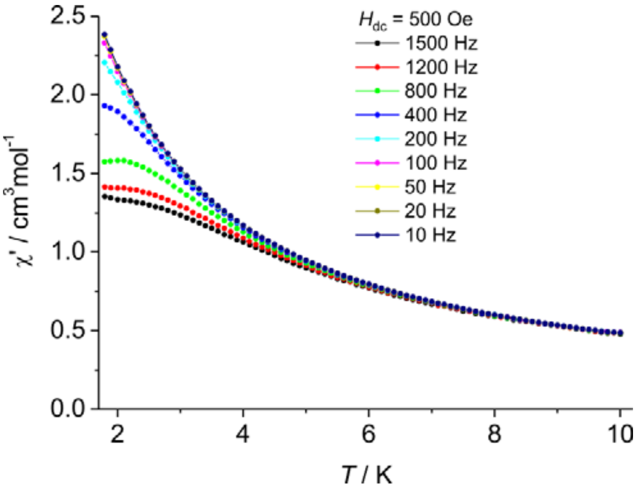

Figure S7. Temperature dependence of χ' at various frequencies with an applied dc field of H_{dc} = 500 Oe.

S4. EPR measurements

Variable-temperature (290–140 K) electron paramagnetic resonance (EPR) spectra were recorded on a Bruker E500 spectrometer equipped with an X-band microwave source (~9.445 GHz). The spectra were analyzed by visual comparison with a locally developed spectral computer simulation program, as described elsewhere.[9]

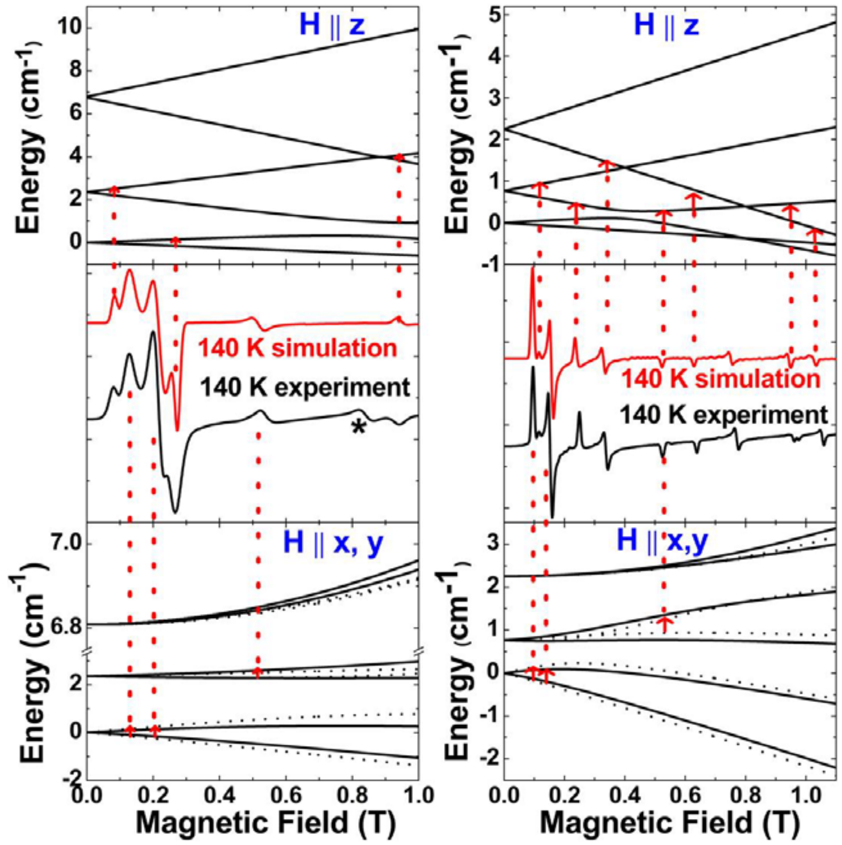

Figure S8. Experimental and simulated spectra of **2** (left) and **3** (right) at 140 K. Also shown are the energy-level diagrams with the magnetic field oriented parallel (H ‖ z) and perpendicular (H ‖ x,y) to the principal symmetry axis of the molecule. The red arrows mark the EPR transition assignments. For **2** the * indicates a peak, which could not be simulated, but could be tentatively ascribed to level-crossing effects.

S5. Theoretical calculations

The complexes **1**, **2** and **3** were optimized at different spin states using DFT functional BP86 which uses the exchange functional of Becke in conjunction with the Perdew's correlation functional (BP86)[10] using Gaussian 09 program package.[11] The double ζ-quality basis set augmented by one set of polarization functions (def2-SVP) is employed for geometrical optimization.[12] The analytical second derivative of energy were calculated to confirm the stationary point on the potential energy surface. The electronic energy of the all the optimized geometries at the BP86/def2-SVP level have further calculated using the meta-GGA exchange functional M06[13] with segmented contracted basis set having triple ζ-quality augmented by two sets of polarization functions (def2-TZVPP).[12] The energies at the M06/def2-TZVPP level of theory were corrected by incorporating the zero point energies from the BP86/def2-SVP level of theory. The Molecular orbital (MO) and Natural Bond Order (NBO)[14] analysis were performed at the M06/def2-TZVPP//BP86/def2-SVP level of theory.

Quantum mechanical calculations show that the quintet state of the tetra coordinated complex **1** is lower in energy than the triplet and singlet electronic states by 59.2 and 63.6 kcal/mol, respectively. The sextet state of **2** is more stable by 5.8 and 38.5 kcal/mol whereas the sextet state of **3** is more stable by 33.3 and 66.6 kcal/mol as compared to their quartet and doublet states. The calculated geometrical parameters of **1**, **2** and **3** in their respective high-spin states are also closest to those of the crystal structures (Figures S12-S14, Tables S3-S5). The C-N bond lengths in free cAAC ligand (1.320 Å) as well as in the complexes **3** (1.324 Å) and **1** (1.330 Å) have similar values. In addition, the C-N bond lengths are slightly elongated in complex **2** (1.350 Å) as compared to free cAAC. This is also well supported by the Wiberg bond index of the C–N bonds viz., 1.47, 1.37, 1.50, and 1.50 for complexes **1**, **2, 3,** and free cAAC, respectively (Table S6). These geometrical data suggest that there is no significant $Cr\rightarrow\pi^*_{cAAC}$ back donation in the complexes **1** and **3**, whereas only little $Cr\rightarrow\pi^*_{cAAC}$ back donation is observed in **2**. The geometrical analysis is in line with the following bonding description based on molecular orbital and NBO analyses.[12]

The valence electron (VE) count of Cr(II) in **1**, Cr(I) in **2**, and Cr(I) in **3** are 12, 11, and 9, respectively. In complex **1**, Cr(II) accepts 8 electrons from two Cl^- and two cAAC ligands. The remaining 4 VEs occupy Cr-based d-orbitals and two among them show slight anti-bonding interaction with the Cl^- p-orbitals (SOMO+2 and SOMO+3, Figure S9a). This is consistent with the NBO spin density, localized mainly on the Cr atom (3.84, Figure S9b) as well as the C–N bond lengths and the corresponding Wiberg bond indices (Tables S6). The NBO group charges of the cAAC ligands and the Cr(II) in **1** indicate $_{cAAC}C\rightarrow Cr$ σ-donation by 0.225 e, and the dissociation energy of one $Cr–C_{cAAC}$ bond is 51.1 kcal/mol.

In complex **2**, six VEs of Cr(I) are utilized for σ-bond formation with two cAAC and one Cl^- ligands. The remaining five VEs occupy Cr-based d-orbitals (Figure S10a). SOMO+3 and SOMO+4 indicate $Cr\rightarrow\pi^*_{cAAC}$ back donation with the cAAC ligands oriented perpendicular to the trigonal plane. Hence, the extent of back donation in **2** is larger than in **1**. In addition, SOMO+3 and SOMO+4 show σ- and π-antibonding interaction between Cl^- and Cr(I) respectively. The SOMO+2 also exhibits π-antibonding interaction between Cl^- and Cr(I). As a result of these interactions, the central Cr atom possesses a spin density of only 4.25 (Figure S10b, Table S7). The $Cr\rightarrow\pi^*_{cAAC}$ back donation is supported by the longer C–N and shorter $Cr–C_{cAAC}$ bonds in **2** as compared to **1** and **3**. The NBO group charge of the cAAC ligand is only 0.010 e, indicating significant amount of $Cr\rightarrow\pi^*_{cAAC}$ back donation as compared to **1** (Table S7). However, the bond dissociation energy for one $Cr–C_{cAAC}$ bond in **2** is 48.6 kcal/mol, which is less than in **1**. The weaker $Cr–C_{cAAC}$ bond strength in **2** can be attributed to the lower oxidation state of Cr.

The SOMOs showing the antibonding interaction between Cr and Cl^- in **2** indicate that the Cl^- ligand is susceptible for removal to give complex, **3**. Four VEs of Cr(I) are utilized to form two σ-bonds with two cAAC ligands and the remaining five VEs reside in the Cr-based d-orbitals, where SOMO+2 reflects $Cr\rightarrow\pi^*_{CAAC}$ back donation (Figure S11a). The extent of back donation in **3** is also less than that in **2**. The NBO spin density is mainly localized on Cr (4.65) with only little contribution from the cAAC ligands (Figure S11b). The NBO group charge of the cAAC ligand indicates a similar extent of $_{cAAC}C\rightarrow Cr$ σ-donation (0.225 e) as in **1**. However, the high dissociation energy for one $Cr–C_{cAAC}$ bond in **3** (79.7 kcal/mol) as compared to **1** can be attributed to the higher charge of the complex. The dissociation energies of one $Au–C_{cAAC}$ bond in $Au(cAAC)_2$, of a Cu–cAAC bond in $Cu(cAAC)_2$, and of a $Co–C_{cAAC}$ in $Co(cAAC)_2$ are 45.6, 48.7, and 64.3 kcal/mol, respectively. Note that these molecules show significant amount of π-back donation, a situation very different from the present Cr complexes. Thus, **1**, **2,** and **3** are examples of low-coordinate low-valent Cr mainly stabilized by the σ-donation of the cAAC ligands.

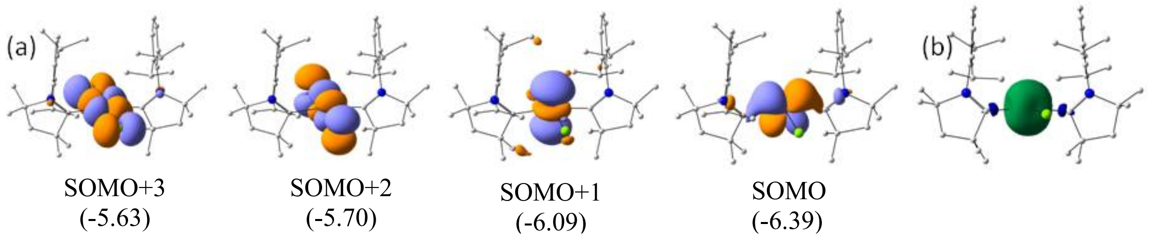

Figure S9. Plots of (a) singly occupied molecular orbitals (energies in eV) of $(cAAC)_2CrCl_2$, **1** and (b) NBO spin density, where the green color corresponds to α-spin density and the blue color corresponds to β-spin density at the M06/def2-TZVPP//BP86/def2-SVP level of theory. Hydrogen atoms are omitted for clarity.

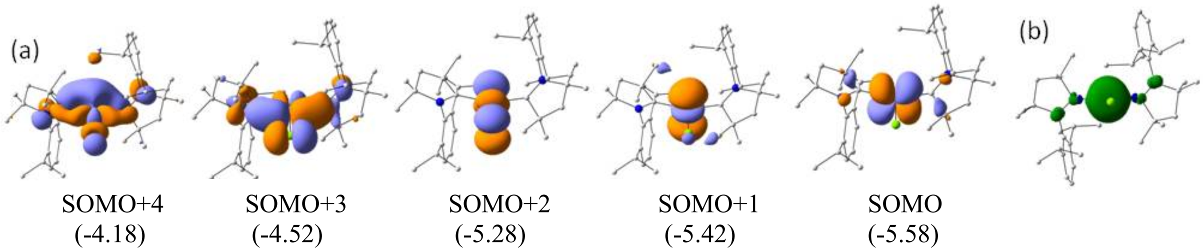

Figure S10. Plots of (a) singly occupied molecular orbitals (energies in eV) of $(cAAC)_2CrCl$, **2** and (b) NBO spin density, where the green color corresponds to α-spin density and the blue color corresponds to β-spin density at the M06/def2-TZVPP//BP86/def2-SVP level of theory. Hydrogen atoms are omitted for clarity**.**

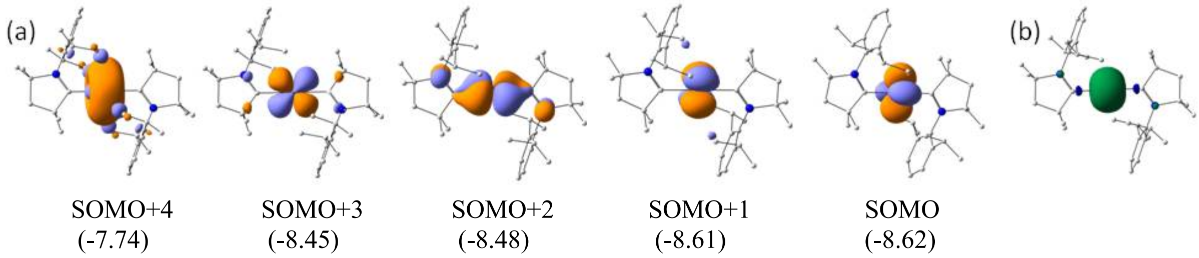

Figure S11. Plots of (a) singly occupied molecular orbitals (energies in eV) of $(cAAC)_2Cr^+$, **3** and (b) NBO spin density, where the green color corresponds to α-spin density and the blue color corresponds to β-spin density at the M06/def2-TZVPP//BP86/def2-SVP level of theory. Hydrogen atoms are omitted for clarity.

Table S2: Optimized Cartesian coordinates of **1**, **2** and **3** in different spin states (Total spin, S) at the BP86/def2-SVP level of theory, the total electronic energy (E^{el}_{M06}) of the molecules at the M06/def2-TZVPP//BP86/def2-SVP level of theory and the total energy including zero point energy (E_{BP86}) at the BP86/def2-SVP level of theory using Gaussian 09 program package. The energies are given in a. u.

1

(Total spin, S = 0)

E^{el}_{M06} = -3634.358197 a. u.

E_{BP86} = -3633.681762 a. u.

Cr	0.035341000	-1.105632000	-0.058201000
N	3.039490000	-0.379664000	0.521336000
Cl	0.106728000	-1.166721000	-2.383331000
C	2.112247000	-1.289997000	0.206378000
C	2.780585000	-2.688077000	0.273437000
H	4.654972000	-3.125999000	1.403931000
C	2.112422000	-3.691008000	1.239831000
H	1.046404000	-3.849149000	0.983304000
H	2.138707000	-3.351558000	2.292709000
H	2.635648000	-4.668548000	1.167243000
H	3.278828000	-4.243376000	-1.181609000
C	5.569515000	-0.199593000	0.970008000
H	6.390319000	-0.670386000	1.549580000
H	5.499249000	0.859936000	1.289273000
H	5.856126000	-0.227999000	-0.097092000
H	4.793898000	-1.536935000	3.280523000
C	4.260669000	-2.391920000	0.672818000
H	4.915953000	-2.438700000	-0.223019000
C	2.718432000	-3.283687000	-1.158308000
H	1.673477000	-3.468489000	-1.475470000
H	3.165665000	-2.601213000	-1.909177000
C	3.994398000	-0.965879000	2.764357000
H	3.017592000	-1.431864000	3.001599000
H	3.995625000	0.060331000	3.178797000
C	4.271945000	-0.963339000	1.246271000
C	2.962150000	1.040995000	0.190688000
C	3.525756000	1.456530000	-1.059403000
C	3.511334000	2.830566000	-1.373954000
H	3.935895000	3.162058000	-2.334352000
C	2.973338000	3.779309000	-0.497061000
H	2.980852000	4.848702000	-0.762060000
C	2.418659000	3.355640000	0.713711000
H	1.980830000	4.100290000	1.396991000
C	2.393009000	1.992943000	1.086041000
C	4.132920000	0.501574000	-2.094455000
H	4.200239000	-0.503030000	-1.629839000
C	3.228746000	0.365612000	-3.338401000
H	3.683885000	-0.337965000	-4.068159000
H	3.108911000	1.344537000	-3.850659000
H	2.221963000	-0.016591000	-3.075307000
C	5.557360000	0.922063000	-2.522477000
H	6.011486000	0.137482000	-3.163605000
H	6.231344000	1.092230000	-1.658180000
H	5.544040000	1.858570000	-3.118829000
C	1.721979000	1.637456000	2.412206000
H	1.732605000	0.535777000	2.527218000
C	2.454160000	2.269721000	3.616336000
H	3.535800000	2.020705000	3.632291000
H	2.008625000	1.915143000	4.569486000
H	2.375246000	3.377901000	3.605270000
C	0.238884000	2.062342000	2.409241000
H	-0.253036000	1.741487000	3.349488000
H	-0.317978000	1.595237000	1.571224000
H	0.125587000	3.163864000	2.323807000
N	-3.025749000	-0.409071000	-0.556857000
Cl	-0.077802000	-1.447661000	2.238593000
C	-2.035650000	-1.282384000	-0.344553000
C	-2.612928000	-2.696458000	-0.578538000
C	-3.824396000	-2.443979000	-1.511160000
H	-4.654980000	-3.157318000	-1.330453000
H	-3.499145000	-2.573223000	-2.563995000
H	-0.779480000	-3.906040000	-0.499127000
H	-2.165475000	-4.700805000	-1.303504000
C	-5.560356000	-0.859528000	-0.458960000
H	-6.404310000	-1.252103000	-1.062500000
H	-5.787778000	0.197875000	-0.218453000
H	-5.526922000	-1.438202000	0.483033000
C	-1.641366000	-3.728919000	-1.177578000
H	-1.242419000	-3.399298000	-2.155528000
C	-3.050341000	-3.214451000	0.822237000
H	-2.196855000	-3.197534000	1.530296000
H	-3.424078000	-4.256388000	0.729816000
H	-3.860358000	-2.601175000	1.263749000
C	-4.477584000	-0.245373000	-2.617033000
H	-5.311615000	-0.729652000	-3.166338000
H	-3.573769000	-0.295344000	-3.254119000
H	-4.750366000	0.817600000	-2.460565000
C	-4.269099000	-0.979195000	-1.283618000
C	-3.014459000	0.994614000	-0.135609000
C	-3.556487000	1.328437000	1.149117000
C	-3.638661000	2.690227000	1.508058000
H	-4.051766000	2.954934000	2.493927000
C	-3.209158000	3.706809000	0.649298000
H	-3.296189000	4.763553000	0.948618000
C	-2.647835000	3.363967000	-0.583406000
H	-2.277113000	4.159921000	-1.248078000
C	-2.524998000	2.020635000	-1.002114000
C	-4.021536000	0.309479000	2.196626000
H	-4.003038000	-0.691395000	1.727095000
C	-3.052639000	0.253641000	3.397433000
H	-3.409655000	-0.492134000	4.139534000
H	-2.994701000	1.236364000	3.912797000
H	-2.033798000	-0.046590000	3.083239000
C	-5.458609000	0.579049000	2.696044000
H	-5.797940000	-0.251561000	3.350254000
H	-6.188767000	0.681616000	1.868543000
H	-5.511340000	1.507924000	3.302271000
C	-1.794218000	1.773696000	-2.323631000
H	-1.771320000	0.682927000	-2.518004000
C	-2.460083000	2.479741000	-3.524662000

H	-3.526131000	2.202321000	-3.645274000
H	-1.931608000	2.211830000	-4.463622000
H	-2.413420000	3.585569000	-3.428037000
C	-0.322370000	2.228462000	-2.207512000
H	0.233308000	1.965890000	-3.130319000
H	0.197091000	1.733813000	-1.362101000
H	-0.245350000	3.326643000	-2.058710000

1

(Total spin, S = 1)

E^{el}_{M06} = -3634.368474 a. u.

E_{BP86} = -3633.694776 a. u.

Cr	0.039453000	-1.118359000	-0.057392000
N	3.037952000	-0.375855000	0.524000000
Cl	0.105546000	-1.181276000	-2.381577000
C	2.112484000	-1.290409000	0.207506000
C	2.783651000	-2.686639000	0.277801000
H	4.661141000	-3.118365000	1.405925000
C	2.117685000	-3.686959000	1.248943000
H	1.051834000	-3.848594000	0.993627000
H	2.143664000	-3.343282000	2.300371000
H	2.642545000	-4.663911000	1.180497000
H	3.285628000	-4.248298000	-1.169855000
C	5.564540000	-0.188715000	0.977316000
H	6.386193000	-0.656836000	1.557731000
H	5.490119000	0.870488000	1.296887000
H	5.852547000	-0.215265000	-0.089537000
H	4.790011000	-1.533650000	3.285060000
C	4.262807000	-2.384937000	0.676306000
H	4.917125000	-2.427828000	-0.220447000
C	2.723434000	-3.289522000	-1.150857000
H	1.679008000	-3.478529000	-1.467584000
H	3.169345000	-2.609706000	-1.904908000
C	3.990267000	-0.962561000	2.769290000
H	3.013952000	-1.430263000	3.004975000
H	3.989919000	0.062669000	3.186076000
C	4.268729000	-0.956614000	1.251136000
C	2.957150000	1.042947000	0.189394000
C	3.518351000	1.456999000	-1.062329000
C	3.498497000	2.829871000	-1.381450000
H	3.921718000	3.160061000	-2.342909000
C	2.956916000	3.779291000	-0.507396000
H	2.959912000	4.847828000	-0.775923000
C	2.404796000	3.357463000	0.705176000
H	1.964684000	4.102687000	1.386369000
C	2.385080000	1.995877000	1.082003000
C	4.130154000	0.500565000	-2.093168000
H	4.202860000	-0.501308000	-1.623556000
C	3.227015000	0.353685000	-3.336486000
H	3.685961000	-0.350825000	-4.062921000
H	3.101499000	1.329431000	-3.853425000
H	2.222287000	-0.032788000	-3.071911000
C	5.552401000	0.926730000	-2.522846000
H	6.010738000	0.142040000	-3.160851000
H	6.225204000	1.103611000	-1.658967000
H	5.534329000	1.860888000	-3.122737000
C	1.719679000	1.642246000	2.411637000
H	1.731591000	0.540622000	2.527539000
C	2.458979000	2.275586000	3.610996000
H	3.540725000	2.026806000	3.620741000
H	2.019110000	1.922296000	4.567273000
H	2.379858000	3.383726000	3.598676000
C	0.236876000	2.068026000	2.416927000
H	-0.249906000	1.747542000	3.360006000
H	-0.325146000	1.602365000	1.581901000
H	0.124253000	3.169707000	2.332939000
N	-3.020788000	-0.407819000	-0.556666000
Cl	-0.091805000	-1.447288000	2.238555000
C	-2.031822000	-1.282983000	-0.339463000
C	-2.611651000	-2.697068000	-0.571072000
C	-3.814192000	-2.442202000	-1.514651000
H	-4.646466000	-3.156143000	-1.344148000
H	-3.478261000	-2.568306000	-2.564525000
H	-0.784906000	-3.916915000	-0.473732000
H	-2.168814000	-4.706542000	-1.285111000
C	-5.555274000	-0.859627000	-0.467536000
H	-6.396873000	-1.253613000	-1.073440000
H	-5.784640000	0.197749000	-0.228823000
H	-5.524349000	-1.437181000	0.475205000
C	-1.640684000	-3.736934000	-1.159035000
H	-1.233482000	-3.414415000	-2.135930000
C	-3.064727000	-3.211112000	0.826147000
H	-2.216361000	-3.205741000	1.540319000
H	-3.450068000	-4.248522000	0.730201000
H	-3.870471000	-2.588975000	1.262851000
C	-4.466733000	-0.244638000	-2.621868000
H	-5.299464000	-0.729197000	-3.172900000
H	-3.561572000	-0.294451000	-3.256988000
H	-4.740203000	0.818277000	-2.466186000
C	-4.260883000	-0.978010000	-1.287757000
C	-3.009159000	0.996217000	-0.137659000
C	-3.553654000	1.333346000	1.145224000
C	-3.633885000	2.695805000	1.502100000
H	-4.049047000	2.962746000	2.486509000
C	-3.200018000	3.710299000	0.643118000
H	-3.285420000	4.767630000	0.940840000
C	-2.636664000	3.364560000	-0.587843000
H	-2.263007000	4.158831000	-1.252892000
C	-2.515780000	2.020370000	-1.004368000
C	-4.024576000	0.317092000	2.192717000
H	-4.004600000	-0.684665000	1.725154000
C	-3.061726000	0.262922000	3.398421000
H	-3.422945000	-0.481099000	4.140274000
H	-3.005608000	1.246585000	3.912179000
H	-2.041715000	-0.038621000	3.089489000
C	-5.463894000	0.589036000	2.684413000
H	-5.807330000	-0.239800000	3.338742000
H	-6.189785000	0.690360000	1.853019000
H	-5.518894000	1.519373000	3.288192000
C	-1.783854000	1.770203000	-2.324618000
H	-1.763290000	0.679169000	-2.517360000
C	-2.446842000	2.476058000	-3.527415000
H	-3.513414000	2.201025000	-3.648754000
H	-1.918084000	2.205638000	-4.465514000
H	-2.397673000	3.581900000	-3.432271000
C	-0.311148000	2.221825000	-2.208032000
H	0.245095000	1.954989000	-3.129292000
H	0.206041000	1.729626000	-1.359997000
H	-0.231661000	3.320330000	-2.063076000

1

(Total spin, S = 2)

E^{el}_{M06} = -3634.462786 a. u.

E_{BP86} = -3633.732928 a. u.

Cr	0.036322000	-1.102172000	-0.061223000
N	3.087398000	-0.382394000	0.507104000
Cl	0.079837000	-1.148594000	-2.405799000
C	2.161225000	-1.288873000	0.208184000
C	2.818167000	-2.687174000	0.277067000
H	4.704487000	-3.129433000	1.386329000
C	2.151686000	-3.678584000	1.255915000
H	1.080449000	-3.823127000	1.014608000
H	2.195954000	-3.333994000	2.306485000
H	2.663678000	-4.661782000	1.180176000
H	3.270437000	-4.253541000	-1.180321000
C	5.629569000	-0.209502000	0.904378000
H	6.459811000	-0.678845000	1.471510000
H	5.569822000	0.852921000	1.216015000
H	5.894520000	-0.248854000	-0.167902000
H	4.892653000	-1.514906000	3.243699000
C	4.306100000	-2.399562000	0.653379000
H	4.948531000	-2.459971000	-0.250853000
C	2.726987000	-3.284440000	-1.153249000
H	1.674590000	-3.450313000	-1.456393000
H	3.176878000	-2.611772000	-1.911413000
C	4.083601000	-0.950543000	2.735378000
H	3.111915000	-1.415288000	2.995183000
H	4.091136000	0.080540000	3.137226000
C	4.335673000	-0.965991000	1.213568000
C	3.003821000	1.040562000	0.180976000
C	3.548522000	1.456827000	-1.076688000
C	3.530265000	2.831720000	-1.387323000
H	3.939426000	3.165150000	-2.353632000
C	3.006498000	3.778396000	-0.499777000
H	3.010889000	4.848453000	-0.762053000
C	2.468492000	3.352068000	0.717551000
H	2.039891000	4.095090000	1.408342000
C	2.446355000	1.988526000	1.087124000
C	4.135885000	0.503661000	-2.124870000
H	4.210277000	-0.502902000	-1.665404000
C	3.208854000	0.373857000	-3.352708000
H	3.647659000	-0.330948000	-4.091218000
H	3.086077000	1.354327000	-3.861308000
H	2.204070000	-0.002798000	-3.073806000
C	5.553040000	0.923588000	-2.577139000
H	5.995641000	0.139120000	-3.226349000
H	6.241950000	1.093720000	-1.724701000
H	5.529184000	1.860090000	-3.173116000
C	1.788024000	1.630271000	2.419181000
H	1.797961000	0.528413000	2.532938000
C	2.528910000	2.262602000	3.617936000
H	3.611638000	2.017738000	3.624794000
H	2.092500000	1.904644000	4.573997000
H	2.446011000	3.370516000	3.609969000
C	0.304301000	2.053151000	2.428154000
H	-0.178128000	1.733266000	3.373620000
H	-0.256258000	1.578863000	1.597354000
H	0.188459000	3.154246000	2.339755000
N	-3.071831000	-0.413842000	-0.543602000
Cl	-0.045095000	-1.431896000	2.257449000
C	-2.083272000	-1.282759000	-0.348040000
C	-2.650763000	-2.696272000	-0.581995000
C	-3.891101000	-2.452390000	-1.479297000
H	-4.714823000	-3.163878000	-1.264316000
H	-3.600130000	-2.593479000	-2.540539000
H	-0.807557000	-3.891637000	-0.553733000
H	-2.203684000	-4.685966000	-1.343854000
C	-5.606683000	-0.859330000	-0.408145000
H	-6.460952000	-1.247501000	-0.999869000
H	-5.825550000	0.198727000	-0.162756000
H	-5.561644000	-1.439969000	0.532288000
C	-1.683163000	-3.712759000	-1.213457000
H	-1.303037000	-3.363393000	-2.192251000
C	-3.039874000	-3.222049000	0.831016000
H	-2.168621000	-3.181706000	1.516602000
H	-3.390130000	-4.272856000	0.748859000
H	-3.853040000	-2.627109000	1.292087000
C	-4.549321000	-0.252361000	-2.583467000
H	-5.390206000	-0.735169000	-3.123444000
H	-3.652174000	-0.305449000	-3.229783000
H	-4.817332000	0.811585000	-2.425410000
C	-4.329213000	-0.985572000	-1.251906000
C	-3.060252000	0.991775000	-0.124464000
C	-3.593895000	1.324349000	1.163329000
C	-3.686828000	2.687118000	1.516047000
H	-4.093717000	2.952691000	2.504194000
C	-3.273247000	3.703318000	0.648858000
H	-3.368600000	4.760635000	0.943568000
C	-2.715030000	3.359967000	-0.585199000
H	-2.353775000	4.155927000	-1.255039000
C	-2.582333000	2.015824000	-0.998388000
C	-4.029252000	0.304075000	2.222178000
H	-4.015245000	-0.698245000	1.755044000
C	-3.029390000	0.259382000	3.397941000
H	-3.360851000	-0.487434000	4.150719000
H	-2.967418000	1.244436000	3.908363000
H	-2.015740000	-0.032642000	3.059170000
C	-5.455676000	0.563691000	2.755549000
H	-5.771173000	-0.267276000	3.421096000
H	-6.207244000	0.656819000	1.946125000
H	-5.501446000	1.494046000	3.360015000
C	-1.850463000	1.766073000	-2.318527000
H	-1.818322000	0.674332000	-2.507481000
C	-2.519046000	2.461902000	-3.523801000
H	-3.583289000	2.177415000	-3.644570000
H	-1.987508000	2.192003000	-4.460399000
H	-2.479204000	3.568548000	-3.433203000
C	-0.381267000	2.230016000	-2.201318000
H	0.176767000	1.966682000	-3.122296000
H	0.138272000	1.738790000	-1.354099000
H	-0.311484000	3.329202000	-2.055494000

2

(Total spin, S = $1/2$)

E^{el}_{M06} = -3174.12682 a. u.

E_{BP86} = -3173.530447 a. u.

Cr	-0.366858000	-1.009913000	-0.339712000
Cl	-0.518877000	-3.174033000	0.297740000
N	2.578144000	-0.040775000	-0.894858000
N	-2.450736000	0.542093000	0.877853000
C	1.355707000	-0.687999000	-1.010538000
C	-1.207347000	-0.052949000	1.083152000
C	1.390519000	-1.495748000	-2.364860000
C	-0.921197000	0.017869000	2.594386000
C	2.397480000	-0.639511000	-3.175987000
H	2.905388000	-1.228710000	-3.968482000
H	1.850215000	0.186714000	-3.678066000
C	-1.775905000	1.239880000	3.033239000
H	-2.108595000	1.165386000	4.089641000
H	-1.159195000	2.159643000	2.949012000
C	-2.979290000	1.340680000	2.059082000
C	3.408048000	-0.034664000	-2.170348000
C	0.021403000	-1.564655000	-3.084558000
H	0.136061000	-2.049386000	-4.078915000
H	-0.421380000	-0.561175000	-3.249979000
H	-0.710759000	-2.194104000	-2.527085000
C	0.559819000	0.206295000	2.954856000
H	0.683205000	0.266853000	4.057589000
H	0.984810000	1.126403000	2.510779000
H	1.163658000	-0.647194000	2.592641000
C	1.898809000	-2.950066000	-2.192010000
H	1.985073000	-3.439200000	-3.186872000
H	1.196669000	-3.534986000	-1.564922000
H	2.893814000	-2.997614000	-1.710764000
C	-1.416451000	-1.292556000	3.264651000
H	-1.267084000	-1.237562000	4.364934000
H	-0.862351000	-2.166556000	2.868773000
H	-2.492029000	-1.480554000	3.075827000
C	3.814345000	1.384514000	-2.610386000
H	4.345476000	1.323269000	-3.583358000
H	4.507236000	1.854635000	-1.884162000
H	2.936958000	2.045382000	-2.747544000
C	4.712557000	-0.852305000	-2.040459000
H	5.290262000	-0.775962000	-2.984647000
H	4.537249000	-1.924882000	-1.841691000
H	5.347542000	-0.446482000	-1.226965000
C	4.344220000	1.360444000	2.749658000
H	4.826096000	1.720235000	3.672879000
C	4.354437000	-0.001566000	2.429587000
H	4.841739000	-0.713130000	3.114832000
C	-4.272244000	0.748487000	2.654452000
H	-4.580406000	1.353276000	3.532394000
H	-4.148602000	-0.296938000	2.992508000
H	-5.100407000	0.786939000	1.919863000
C	-3.149291000	0.408129000	-0.388078000
C	3.127622000	0.437677000	0.356817000
C	5.263150000	-2.551063000	1.067635000
H	5.281985000	-3.617516000	0.758402000
H	5.674547000	-2.506223000	2.098646000
H	5.957815000	-1.992204000	0.408122000
C	-4.546000000	0.192721000	-2.855424000
H	-5.094361000	0.112422000	-3.807659000
C	-3.620214000	1.221203000	-2.655385000
H	-3.438853000	1.949309000	-3.462144000
C	-2.911047000	1.352791000	-1.442419000
C	3.761277000	-0.491101000	1.246996000
C	-1.899400000	2.496395000	-1.347102000
H	-1.494497000	2.497374000	-0.317904000
C	3.820985000	-2.001580000	1.001895000
H	3.430751000	-2.187430000	-0.015429000
C	-4.083358000	-0.664774000	-0.598115000
C	3.074276000	1.825397000	0.702182000
C	-4.760873000	-0.738376000	-1.834599000
H	-5.478936000	-1.557583000	-1.996999000
C	-2.539117000	3.874982000	-1.618561000
H	-1.803201000	4.685838000	-1.433034000
H	-3.420151000	4.060999000	-0.972624000
H	-2.872017000	3.972297000	-2.673512000
C	-4.402583000	-1.760402000	0.425251000
H	-3.766035000	-1.584281000	1.314376000
C	2.302045000	2.874528000	-0.101330000
H	1.928482000	2.377668000	-1.016710000
C	-0.708496000	2.262972000	-2.299185000
H	-0.003696000	3.120080000	-2.262437000
H	-1.040941000	2.148559000	-3.353009000
H	-0.144345000	1.349277000	-2.008006000
C	2.908365000	-2.781042000	1.973181000
H	1.841295000	-2.529356000	1.816535000
H	3.173330000	-2.578593000	3.033028000
H	3.012012000	-3.873421000	1.802364000
C	-5.887010000	-1.730426000	0.858222000
H	-6.063480000	-2.457182000	1.679057000
H	-6.552989000	-2.018478000	0.017193000
H	-6.214205000	-0.732651000	1.210961000
C	1.066279000	3.363352000	0.685375000
H	0.504118000	4.121539000	0.099415000
H	1.358730000	3.836872000	1.646866000
H	0.382407000	2.521208000	0.911674000
C	-4.061379000	-3.167857000	-0.111833000
H	-4.266359000	-3.930051000	0.669404000
H	-2.992029000	-3.254630000	-0.383606000
H	-4.681497000	-3.427288000	-0.996515000
C	3.168759000	4.084468000	-0.511040000
H	2.596397000	4.764056000	-1.177717000
H	4.091935000	3.783285000	-1.044580000
H	3.477200000	4.681855000	0.373085000
C	3.700682000	2.257178000	1.891040000
H	3.665779000	3.326296000	2.155092000
C	-3.277635000	2.805051000	1.683960000
H	-3.637918000	3.348037000	2.582518000
H	-4.073113000	2.873594000	0.913276000
H	-2.376852000	3.331028000	1.313786000

2

(Total spin, S = $3/2$)

E^{el}_{M06} = -3174.178858 a. u.

E_{BP86} = -3173.561162 a. u.

Cr	-0.000052000	-1.434960000	-0.000080000
Cl	-0.000221000	-3.707707000	-0.000094000
N	-2.401298000	0.273267000	0.982373000
N	2.401257000	0.273353000	-0.982329000
C	-1.174240000	-0.328111000	1.121926000
C	1.174167000	-0.327948000	-1.121936000
C	-0.925206000	-0.512319000	2.649053000
C	0.925140000	-0.512011000	-2.649083000

C	-1.941520000	0.465221000	3.291519000
H	-2.287563000	0.129465000	4.291117000
H	-1.457878000	1.456264000	3.425938000
C	1.941566000	0.465466000	-3.291478000
H	2.287620000	0.129708000	-4.291072000
H	1.458019000	1.456557000	-3.425896000
C	3.112580000	0.609745000	-2.295051000
C	-3.112558000	0.609636000	2.295137000
C	0.520247000	-0.193353000	3.075531000
H	0.641508000	-0.343152000	4.170203000
H	0.805406000	0.847742000	2.835942000
H	1.245607000	-0.860324000	2.564288000
C	-0.520275000	-0.192873000	-3.075556000
H	-0.641575000	-0.342709000	-4.170218000
H	-0.805288000	0.848273000	-2.836014000
H	-1.245717000	-0.859715000	-2.564260000
C	-1.216268000	-1.983939000	3.062879000
H	-1.121321000	-2.087771000	4.165750000
H	-0.504007000	-2.684204000	2.581221000
H	-2.231791000	-2.319979000	2.776178000
C	1.216064000	-1.983641000	-3.062978000
H	1.121128000	-2.087417000	-4.165855000
H	0.503724000	-2.683863000	-2.581369000
H	2.231551000	-2.319792000	-2.776274000
C	-3.697499000	2.030977000	2.320680000
H	-4.201288000	2.192148000	3.296264000
H	-4.457206000	2.175902000	1.526296000
H	-2.917611000	2.808242000	2.216843000
C	-4.274926000	-0.364352000	2.580923000
H	-4.703971000	-0.142992000	3.580048000
H	-3.960118000	-1.423695000	2.574714000
H	-5.084957000	-0.235804000	1.835776000
C	-4.549297000	0.886543000	-2.678053000
H	-5.132195000	1.055750000	-3.597513000
C	-4.541923000	-0.370300000	-2.065882000
H	-5.116690000	-1.192864000	-2.520080000
C	4.274889000	-0.364310000	-2.580855000
H	4.704036000	-0.142866000	-3.579917000
H	3.959986000	-1.423626000	-2.574802000
H	5.084867000	-0.235925000	-1.835623000
C	3.080359000	0.457589000	0.288932000
C	-3.080342000	0.457830000	-0.288875000
C	-5.271021000	-2.528200000	0.016424000
H	-5.236421000	-3.537478000	0.477888000
H	-5.912299000	-2.604237000	-0.887509000
H	-5.778932000	-1.846404000	0.726008000
C	4.549417000	0.885510000	2.678180000
H	5.132351000	1.054407000	3.597674000
C	3.792078000	1.919574000	2.118847000
H	3.771328000	2.902643000	2.615551000
C	3.045703000	1.735153000	0.935888000
C	-3.815006000	-0.620922000	-0.881261000
C	2.197828000	2.917883000	0.456650000
H	1.757548000	2.640553000	-0.520283000
C	-3.844671000	-2.058241000	-0.347479000
H	-3.213276000	-2.101733000	0.562734000
C	3.814791000	-0.621435000	0.881093000
C	-3.045385000	1.735504000	-0.935603000
C	4.541749000	-0.371220000	2.065774000
H	5.116320000	-1.194005000	2.519817000
C	3.020346000	4.212895000	0.276000000
H	2.399606000	5.001855000	-0.199128000
H	3.917929000	4.060103000	-0.355025000
H	3.364765000	4.616816000	1.251460000
C	3.844153000	-2.058633000	0.346968000
H	3.212832000	-2.101739000	-0.563313000
C	-2.197240000	2.917947000	-0.456137000
H	-1.757249000	2.640436000	0.520874000
C	1.020522000	3.180300000	1.420420000
H	0.439538000	4.068728000	1.095350000
H	1.378905000	3.377894000	2.453111000
H	0.334075000	2.311716000	1.450328000
C	-3.233062000	-3.042689000	-1.368914000
H	-2.193830000	-2.768073000	-1.635608000
H	-3.836111000	-3.086892000	-2.300893000
H	-3.190225000	-4.064850000	-0.940554000
C	5.270418000	-2.528819000	-0.016974000
H	5.235618000	-3.537961000	-0.478722000
H	5.911628000	-2.605252000	0.886974000
H	5.778523000	-1.846939000	-0.726341000
C	-1.019647000	3.180030000	-1.419649000
H	-0.438464000	4.068274000	-1.094434000
H	-1.377748000	3.377757000	-2.452411000
H	-0.333457000	2.311236000	-1.449425000
C	3.232207000	-3.043207000	1.368081000
H	3.189323000	-4.065277000	0.939506000
H	2.192952000	-2.768512000	1.634615000
H	3.835060000	-3.087682000	2.300174000
C	-3.019391000	4.213210000	-0.275606000
H	-2.398504000	5.001954000	0.199689000
H	-3.917155000	4.060673000	0.355221000
H	-3.363477000	4.617283000	-1.251120000
C	-3.791701000	1.920318000	-2.118537000
H	-3.770712000	2.903472000	-2.615064000
C	3.697626000	2.031046000	-2.320485000
H	4.201500000	2.192233000	-3.296023000
H	4.457282000	2.175876000	-1.526034000
H	2.917787000	2.808362000	-2.216667000

2

(Total spin, S = $5/2$)

E^{el}_{M06} = -3174.188122 a. u.

E_{BP86} = -3173.563902 a. u.

Cr	0.000061000	-0.873476000	-0.000089000
Cl	0.000184000	-3.205744000	-0.000366000
N	-2.857698000	0.201855000	0.922503000
N	2.857749000	0.202071000	-0.922461000
C	-1.624631000	-0.286068000	1.177352000
C	1.624694000	-0.285793000	-1.177470000
C	-1.523215000	-0.499266000	2.704202000
C	1.523324000	-0.498626000	-2.704371000
C	-2.684088000	0.357517000	3.278286000
H	-3.123741000	-0.075425000	4.200461000
H	-2.299544000	1.366410000	3.540121000
C	2.684286000	0.358210000	-3.278211000
H	3.123958000	-0.074561000	-4.200456000
H	2.299807000	1.367179000	-3.539854000
C	3.735393000	0.492868000	-2.152217000
C	-3.735251000	0.492466000	2.152371000

C	-0.156483000	-0.066445000	3.269311000
H	-0.109487000	-0.257332000	4.363391000
H	0.034752000	1.012096000	3.101577000
H	0.670594000	-0.635595000	2.793006000
C	0.156647000	-0.065560000	-3.269405000
H	0.109602000	-0.256266000	-4.363512000
H	-0.034469000	1.012976000	-3.101495000
H	-0.670478000	-0.634695000	-2.793162000
C	-1.714723000	-2.011871000	3.002931000
H	-1.635843000	-2.189653000	4.097224000
H	-0.949139000	-2.616965000	2.476748000
H	-2.703354000	-2.384686000	2.668991000
C	1.714710000	-2.011178000	-3.003450000
H	1.635845000	-2.188698000	-4.097788000
H	0.949049000	-2.616322000	-2.477441000
H	2.703287000	-2.384172000	-2.669558000
C	-4.348793000	1.900778000	2.111334000
H	-4.957597000	2.053533000	3.026482000
H	-5.018845000	2.031957000	1.237413000
H	-3.574380000	2.690884000	2.089657000
C	-4.888173000	-0.521841000	2.274908000
H	-5.464057000	-0.309529000	3.199248000
H	-4.532835000	-1.567601000	2.329821000
H	-5.587359000	-0.433436000	1.419759000
C	-4.576384000	0.813300000	-2.948917000
H	-5.055837000	0.983279000	-3.926207000
C	-4.619855000	-0.450033000	-2.350840000
H	-5.122618000	-1.277639000	-2.875857000
C	4.888254000	-0.521493000	-2.274935000
H	5.464223000	-0.308957000	-3.199170000
H	4.532848000	-1.567211000	-2.330176000
H	5.587383000	-0.433391000	-1.419709000
C	3.385984000	0.386914000	0.420933000
C	-3.386057000	0.386928000	-0.420813000
C	-5.501972000	-2.687562000	-0.432296000
H	-5.484235000	-3.698588000	0.026078000
H	-5.996947000	-2.787739000	-1.421425000
H	-6.145844000	-2.040475000	0.196376000
C	4.575951000	0.812843000	2.949265000
H	5.055256000	0.982646000	3.926659000
C	3.895232000	1.852035000	2.305786000
H	3.826596000	2.836446000	2.795474000
C	3.283177000	1.667330000	1.048316000
C	-4.026514000	-0.700950000	-1.093966000
C	2.472508000	2.835031000	0.477951000
H	2.145292000	2.556388000	-0.543980000
C	-4.063262000	-2.143356000	-0.577458000
H	-3.572990000	-2.165597000	0.415674000
C	4.026390000	-0.701071000	1.093954000
C	-3.283348000	1.667448000	-1.047990000
C	4.619557000	-0.450375000	2.350952000
H	5.122282000	-1.278056000	2.875887000
C	3.277951000	4.148857000	0.390337000
H	2.683258000	4.932549000	-0.124758000
H	4.229652000	4.023759000	-0.163492000
H	3.528291000	4.542541000	1.398017000
C	4.063269000	-2.143364000	0.577139000
H	3.573030000	-2.165420000	-0.416013000
C	-2.472630000	2.835070000	-0.477543000
H	-2.145278000	2.556252000	0.544297000
C	1.195822000	3.049452000	1.319780000
H	0.609986000	3.909274000	0.933664000
H	1.446129000	3.263592000	2.380594000
H	0.549987000	2.147985000	1.289837000
C	-3.246903000	-3.074742000	-1.500910000
H	-2.182967000	-2.772056000	-1.550249000
H	-3.668400000	-3.094380000	-2.528679000
H	-3.263747000	-4.112542000	-1.107610000
C	5.502040000	-2.687398000	0.431894000
H	5.484431000	-3.698312000	-0.026730000
H	5.996974000	-2.787763000	1.421023000
H	6.145876000	-2.040086000	-0.196585000
C	-1.196066000	3.049664000	-1.319516000
H	-0.610147000	3.909376000	-0.933277000
H	-1.446527000	3.264083000	-2.380237000
H	-0.550241000	2.148184000	-1.289930000
C	3.246966000	-3.075044000	1.500340000
H	3.264006000	-4.112764000	1.106833000
H	2.182983000	-2.772521000	1.549657000
H	3.668388000	-3.094842000	2.528138000
C	-3.278073000	4.148875000	-0.389627000
H	-2.683304000	4.932495000	0.125487000
H	-4.229693000	4.023704000	0.164325000
H	-3.528560000	4.542687000	-1.397223000
C	-3.895600000	1.852383000	-2.305330000
H	-3.827044000	2.836893000	-2.794833000
C	4.349049000	1.901121000	-2.110849000
H	4.958061000	2.053964000	-3.025843000
H	5.018927000	2.032094000	-1.236764000
H	3.574709000	2.691297000	-2.089209000

3

(Total spin, S = $1/2$)

E^{el}_{M06} = -2713.661168 a. u.

E_{BP86} = -2713.176737 a. u.

Cr	0.000000000	0.000000000	0.000000000
N	-2.098163000	-1.347547000	1.600012000
C	-2.048411000	-0.129593000	2.391960000
C	-1.330594000	-0.093650000	3.625568000
C	-1.331425000	-1.461427000	0.498461000
C	-2.728197000	1.030539000	1.901807000
C	-3.150867000	-2.442296000	1.816860000
C	-1.746151000	-2.750913000	-0.233213000
C	-3.364059000	1.074922000	0.511554000
H	-3.490566000	0.034495000	0.162877000
C	-1.382341000	1.094263000	4.386703000
H	-0.844922000	1.138633000	5.346875000
C	-0.428104000	-1.231064000	4.108405000
H	-0.577599000	-2.091737000	3.425869000
C	-2.737816000	2.189744000	2.703695000
H	-3.266700000	3.087802000	2.349175000
C	-4.754144000	1.742112000	0.482849000
H	-4.707886000	2.822810000	0.730612000
H	-5.448288000	1.261783000	1.201386000
H	-5.201788000	1.659721000	-0.529032000
C	-2.427122000	1.770697000	-0.492298000
H	-1.363912000	1.378507000	-0.513436000
H	-2.297319000	2.846739000	-0.255726000
H	-2.804280000	1.684200000	-1.531035000
C	-2.091136000	2.218672000	3.946926000

H	-2.125700000	3.128048000	4.566994000
C	1.054432000	-0.811220000	3.990252000
H	1.298589000	-0.481797000	2.958822000
H	1.724772000	-1.656984000	4.250841000
H	1.294281000	0.029567000	4.674503000
C	-0.740799000	-1.692465000	5.546714000
H	-1.801070000	-1.988665000	5.670893000
H	-0.528209000	-0.893888000	6.287707000
H	-0.109537000	-2.563806000	5.818217000
C	-0.530702000	-3.612398000	-0.631436000
H	-0.869856000	-4.547907000	-1.124453000
H	0.071848000	-3.898770000	0.254892000
H	0.130199000	-3.078435000	-1.346149000
C	-2.517654000	-2.362929000	-1.521837000
H	-2.852556000	-3.278083000	-2.054265000
H	-1.869520000	-1.787317000	-2.214652000
H	-3.416204000	-1.749016000	-1.310194000
C	-3.147006000	-2.979621000	3.255120000
H	-2.169329000	-3.415086000	3.536656000
H	-3.906923000	-3.782824000	3.340717000
H	-3.414626000	-2.191611000	3.987932000
C	-4.566770000	-1.916405000	1.501225000
H	-4.829727000	-1.052734000	2.145341000
H	-5.304958000	-2.718271000	1.704888000
H	-4.687100000	-1.618845000	0.441957000
C	-2.651674000	-3.493631000	0.795841000
H	-3.495973000	-4.024934000	0.311929000
H	-2.049725000	-4.259876000	1.327376000
N	2.098163000	1.347547000	-1.600012000
C	2.048411000	0.129593000	-2.391960000
C	1.330594000	0.093650000	-3.625568000
C	1.331425000	1.461427000	-0.498461000
C	2.728197000	-1.030539000	-1.901807000
C	3.150867000	2.442296000	-1.816860000
C	1.746151000	2.750913000	0.233213000
C	3.364059000	-1.074922000	-0.511554000
H	3.490566000	-0.034495000	-0.162877000
C	1.382341000	-1.094263000	-4.386703000
H	0.844922000	-1.138633000	-5.346875000
C	0.428104000	1.231064000	-4.108405000
H	0.577599000	2.091737000	-3.425869000
C	2.737816000	-2.189744000	-2.703695000
H	3.266700000	-3.087802000	-2.349175000
C	4.754144000	-1.742112000	-0.482849000
H	4.707886000	-2.822810000	-0.730612000
H	5.448288000	-1.261783000	-1.201386000
H	5.201788000	-1.659721000	0.529032000
C	2.427122000	-1.770697000	0.492298000
H	1.363912000	-1.378507000	0.513436000
H	2.297319000	-2.846739000	0.255726000
H	2.804280000	-1.684200000	1.531035000
C	2.091136000	-2.218672000	-3.946926000
H	2.125700000	-3.128048000	-4.566994000
C	-1.054432000	0.811220000	-3.990252000
H	-1.298589000	0.481797000	-2.958822000
H	-1.724772000	1.656984000	-4.250841000
H	-1.294281000	-0.029567000	-4.674503000
C	0.740799000	1.692465000	-5.546714000
H	1.801070000	1.988665000	-5.670893000
H	0.528209000	0.893888000	-6.287707000
H	0.109537000	2.563806000	-5.818217000
C	0.530702000	3.612398000	0.631436000
H	0.869856000	4.547907000	1.124453000
H	-0.071848000	3.898770000	-0.254892000
H	-0.130199000	3.078435000	1.346149000
C	2.517654000	2.362929000	1.521837000
H	2.852556000	3.278083000	2.054265000
H	1.869520000	1.787317000	2.214652000
H	3.416204000	1.749016000	1.310194000
C	3.147006000	2.979621000	-3.255120000
H	2.169329000	3.415086000	-3.536656000
H	3.906923000	3.782824000	-3.340717000
H	3.414626000	2.191611000	-3.987932000
C	4.566770000	1.916405000	-1.501225000
H	4.829727000	1.052734000	-2.145341000
H	5.304958000	2.718271000	-1.704888000
H	4.687100000	1.618845000	-0.441957000
C	2.651674000	3.493631000	-0.795841000
H	3.495973000	4.024934000	-0.311929000
H	2.049725000	4.259876000	-1.327376000

3

(Total spin, S = $3/2$)

E^{el}_{M06} = -2713.714225 a. u.

E_{BP86} = -2713.206922 a. u.

Cr	0.000000000	0.000000000	0.000000000
N	-2.108129000	-1.348114000	1.601466000
C	-2.058514000	-0.130359000	2.392330000
C	-1.334936000	-0.093677000	3.622817000
C	-1.336474000	-1.465796000	0.501270000
C	-2.737597000	1.031043000	1.903900000
C	-3.159014000	-2.443335000	1.816962000
C	-1.748626000	-2.755593000	-0.229510000
C	-3.378990000	1.077073000	0.516209000
H	-3.500777000	0.037184000	0.163979000
C	-1.382169000	1.094594000	4.383706000
H	-0.841127000	1.138718000	5.341881000
C	-0.432951000	-1.232595000	4.103159000
H	-0.585978000	-2.092915000	3.420998000
C	-2.742498000	2.190768000	2.705248000
H	-3.271318000	3.089466000	2.352114000
C	-4.772833000	1.737082000	0.496335000
H	-4.730979000	2.817029000	0.748112000
H	-5.461054000	1.250465000	1.216314000
H	-5.224732000	1.656086000	-0.513762000
C	-2.449948000	1.782366000	-0.488489000
H	-1.390276000	1.383377000	-0.522255000
H	-2.315825000	2.855858000	-0.243092000
H	-2.835858000	1.706043000	-1.524949000
C	-2.091256000	2.219636000	3.946056000
H	-2.122290000	3.129455000	4.565675000
C	1.050328000	-0.816139000	3.981988000
H	1.295830000	-0.489157000	2.950098000
H	1.719068000	-1.663013000	4.243052000
H	1.293243000	0.025188000	4.664492000
C	-0.743041000	-1.693292000	5.542282000
H	-1.803689000	-1.987118000	5.668830000
H	-0.527012000	-0.895162000	6.282784000
H	-0.113136000	-2.566071000	5.812331000
C	-0.531996000	-3.617247000	-0.624486000

H	-0.869085000	-4.554310000	-1.116058000
H	0.069985000	-3.900478000	0.263242000
H	0.129326000	-3.084428000	-1.339852000
C	-2.519377000	-2.372243000	-1.520171000
H	-2.853517000	-3.288768000	-2.050858000
H	-1.871050000	-1.798530000	-2.214557000
H	-3.418241000	-1.757963000	-1.311072000
C	-3.159611000	-2.977032000	3.256749000
H	-2.182717000	-3.411649000	3.542321000
H	-3.919782000	-3.779963000	3.342601000
H	-3.429257000	-2.186939000	3.986619000
C	-4.575117000	-1.921680000	1.494779000
H	-4.843251000	-1.058512000	2.137443000
H	-5.312228000	-2.725349000	1.695326000
H	-4.691347000	-1.624637000	0.434913000
C	-2.654534000	-3.496948000	0.800493000
H	-3.496135000	-4.032739000	0.316802000
H	-2.051257000	-4.259225000	1.336254000
N	2.108129000	1.348114000	-1.601466000
C	2.058514000	0.130359000	-2.392330000
C	1.334936000	0.093677000	-3.622817000
C	1.336474000	1.465796000	-0.501270000
C	2.737597000	-1.031043000	-1.903900000
C	3.159014000	2.443335000	-1.816962000
C	1.748626000	2.755593000	0.229510000
C	3.378990000	-1.077073000	-0.516209000
H	3.500777000	-0.037184000	-0.163979000
C	1.382169000	-1.094594000	-4.383706000
H	0.841127000	-1.138718000	-5.341881000
C	0.432951000	1.232595000	-4.103159000
H	0.585978000	2.092915000	-3.420998000
C	2.742498000	-2.190768000	-2.705248000
H	3.271318000	-3.089466000	-2.352114000
C	4.772833000	-1.737082000	-0.496335000
H	4.730979000	-2.817029000	-0.748112000
H	5.461054000	-1.250465000	-1.216314000
H	5.224732000	-1.656086000	0.513762000
C	2.449948000	-1.782366000	0.488489000
H	1.390276000	-1.383377000	0.522255000
H	2.315825000	-2.855858000	0.243092000
H	2.835858000	-1.706043000	1.524949000
C	2.091256000	-2.219636000	-3.946056000
H	2.122290000	-3.129455000	-4.565675000
C	-1.050328000	0.816139000	-3.981988000
H	-1.295830000	0.489157000	-2.950098000
H	-1.719068000	1.663013000	-4.243052000
H	-1.293243000	-0.025188000	-4.664492000
C	0.743041000	1.693292000	-5.542282000
H	1.803689000	1.987118000	-5.668830000
H	0.527012000	0.895162000	-6.282784000
H	0.113136000	2.566071000	-5.812331000
C	0.531996000	3.617247000	0.624486000
H	0.869085000	4.554310000	1.116058000
H	-0.069985000	3.900478000	-0.263242000
H	-0.129326000	3.084428000	1.339852000
C	2.519377000	2.372243000	1.520171000
H	2.853517000	3.288768000	2.050858000
H	1.871050000	1.798530000	2.214557000
H	3.418241000	1.757963000	1.311072000
C	3.159611000	2.977032000	-3.256749000
H	2.182717000	3.411649000	-3.542321000
H	3.919782000	3.779963000	-3.342601000
H	3.429257000	2.186939000	-3.986619000
C	4.575117000	1.921680000	-1.494779000
H	4.843251000	1.058512000	-2.137443000
H	5.312228000	2.725349000	-1.695326000
H	4.691347000	1.624637000	-0.434913000
C	2.654534000	3.496948000	-0.800493000
H	3.496135000	4.032739000	-0.316802000
H	2.051257000	4.259225000	-1.336254000

3

(Total spin, S = $5/2$)

E^{el}_{M06} = -2713.767321 a. u.

E_{BP86} = -2713.240658 a. u.

Cr	0.000000000	0.000000000	0.000000000
N	-2.373831000	-1.232971000	1.563753000
C	-2.340545000	-0.038987000	2.402769000
C	-1.522476000	-0.047067000	3.571696000
C	-1.534400000	-1.403023000	0.554114000
C	-3.106847000	1.105129000	2.032068000
C	-3.401556000	-2.367850000	1.793753000
C	-1.842089000	-2.741146000	-0.121396000
C	-3.862714000	1.224539000	0.705488000
H	-3.888852000	0.223017000	0.235321000
C	-1.543891000	1.096947000	4.396368000
H	-0.925971000	1.113564000	5.307455000
C	-0.559518000	-1.187073000	3.917280000
H	-0.806235000	-2.051944000	3.269395000
C	-3.085837000	2.217253000	2.900871000
H	-3.674108000	3.111063000	2.641337000
C	-5.319544000	1.700477000	0.877257000
H	-5.372116000	2.742999000	1.254099000
H	-5.885896000	1.062417000	1.585790000
H	-5.849624000	1.680656000	-0.097356000
C	-3.096990000	2.149169000	-0.266097000
H	-2.058807000	1.782729000	-0.435394000
H	-3.024457000	3.184393000	0.128272000
H	-3.606663000	2.194123000	-1.251119000
C	-2.329501000	2.212477000	4.079330000
H	-2.339825000	3.088725000	4.746256000
C	0.891344000	-0.779993000	3.578872000
H	0.988396000	-0.491695000	2.506820000
H	1.592896000	-1.618850000	3.768897000
H	1.222049000	0.086357000	4.189572000
C	-0.668551000	-1.653102000	5.383262000
H	-1.707229000	-1.929884000	5.654464000
H	-0.336301000	-0.867182000	6.092856000
H	-0.022177000	-2.538481000	5.555300000
C	-0.547957000	-3.547400000	-0.358690000
H	-0.789228000	-4.533561000	-0.808582000
H	-0.000295000	-3.730975000	0.588700000
H	0.134577000	-3.015406000	-1.054472000
C	-2.512930000	-2.452213000	-1.489878000
H	-2.751848000	-3.409519000	-1.998626000

H	-1.833970000	-1.875509000	-2.150681000
H	-3.454762000	-1.876738000	-1.386715000
C	-3.462270000	-2.792776000	3.267806000
H	-2.495099000	-3.186172000	3.633414000
H	-4.210835000	-3.604323000	3.371517000
H	-3.781897000	-1.958528000	3.924482000
C	-4.809293000	-1.924189000	1.353631000
H	-5.172404000	-1.067588000	1.955089000
H	-5.512876000	-2.765398000	1.516909000
H	-4.856726000	-1.652538000	0.281570000
C	-2.798975000	-3.459676000	0.876887000
H	-3.589724000	-4.041302000	0.362375000
H	-2.217948000	-4.177355000	1.492735000
N	2.373831000	1.232971000	-1.563753000
C	2.340545000	0.038987000	-2.402769000
C	1.522476000	0.047067000	-3.571696000
C	1.534400000	1.403023000	-0.554114000
C	3.106847000	-1.105129000	-2.032068000
C	3.401556000	2.367850000	-1.793753000
C	1.842089000	2.741146000	0.121396000
C	3.862714000	-1.224539000	-0.705488000
H	3.888852000	-0.223017000	-0.235321000
C	1.543891000	-1.096947000	-4.396368000
H	0.925971000	-1.113564000	-5.307455000
C	0.559518000	1.187073000	-3.917280000
H	0.806235000	2.051944000	-3.269395000
C	3.085837000	-2.217253000	-2.900871000
H	3.674108000	-3.111063000	-2.641337000
C	5.319544000	-1.700477000	-0.877257000
H	5.372116000	-2.742999000	-1.254099000
H	5.885896000	-1.062417000	-1.585790000
H	5.849624000	-1.680656000	0.097356000
C	3.096990000	-2.149169000	0.266097000
H	2.058807000	-1.782729000	0.435394000
H	3.024457000	-3.184393000	-0.128272000
H	3.606663000	-2.194123000	1.251119000
C	2.329501000	-2.212477000	-4.079330000
H	2.339825000	-3.088725000	-4.746256000
C	-0.891344000	0.779993000	-3.578872000
H	-0.988396000	0.491695000	-2.506820000
H	-1.592896000	1.618850000	-3.768897000
H	-1.222049000	-0.086357000	-4.189572000
C	0.668551000	1.653102000	-5.383262000
H	1.707229000	1.929884000	-5.654464000
H	0.336301000	0.867182000	-6.092856000
H	0.022177000	2.538481000	-5.555300000
C	0.547957000	3.547400000	0.358690000
H	0.789228000	4.533561000	0.808582000
H	0.000295000	3.730975000	-0.588700000
H	-0.134577000	3.015406000	1.054472000
C	2.512930000	2.452213000	1.489878000
H	2.751848000	3.409519000	1.998626000
H	1.833970000	1.875509000	2.150681000
H	3.454762000	1.876738000	1.386715000
C	3.462270000	2.792776000	-3.267806000
H	2.495099000	3.186172000	-3.633414000
H	4.210835000	3.604323000	-3.371517000
H	3.781897000	1.958528000	-3.924482000
C	4.809293000	1.924189000	-1.353631000
H	5.172404000	1.067588000	-1.955089000
H	5.512876000	2.765398000	-1.516909000
H	4.856726000	1.652538000	-0.281570000
C	2.798975000	3.459676000	-0.876887000
H	3.589724000	4.041302000	-0.362375000
H	2.217948000	4.177355000	-1.492735000

cAAC

E^{el}_{M06} = -834.695639 a. u.

E_{BP86} = -834.417067 a. u.

N	-0.608634000	-0.055451000	0.063929000
C	-1.341279000	-0.084437000	-1.033221000
C	-2.800132000	-0.188280000	-0.585395000
C	-2.760176000	-0.483796000	0.953744000
H	-3.541160000	0.069109000	1.515338000
H	-2.940997000	-1.564942000	1.130418000
C	-1.340132000	-0.110592000	1.440563000
C	-3.503770000	-1.315654000	-1.369819000
H	-4.564625000	-1.413736000	-1.051555000
H	-3.005163000	-2.294050000	-1.206130000
H	-3.481107000	-1.106341000	-2.458566000
C	-3.489023000	1.160323000	-0.912046000
H	-4.566286000	1.117582000	-0.642219000
H	-3.407667000	1.387313000	-1.994390000
H	-3.033104000	2.007023000	-0.358213000
C	-0.724579000	-1.161198000	2.377069000
H	-1.281670000	-1.167680000	3.337254000
H	0.336488000	-0.932657000	2.606806000
H	-0.780135000	-2.182456000	1.952702000
C	-1.283592000	1.262217000	2.136550000
H	-1.802420000	1.201158000	3.116015000
H	-1.780809000	2.053864000	1.542408000
H	-0.236884000	1.572900000	2.332252000
C	3.645229000	0.197077000	0.018883000
H	4.744805000	0.259480000	0.050085000
C	2.875452000	1.366610000	-0.026652000
H	3.380457000	2.344590000	-0.060703000
C	0.841696000	0.038989000	0.001270000
C	1.169056000	3.811278000	0.511927000
H	0.491593000	4.678334000	0.364334000
H	2.182069000	4.138976000	0.195807000
H	1.211713000	3.593331000	1.599589000
C	1.465361000	1.317365000	-0.053879000
C	0.670281000	2.601197000	-0.299033000
H	-0.378700000	2.410857000	-0.002942000
C	1.606465000	-1.161660000	-0.044159000
C	0.957148000	-2.528471000	-0.272138000
H	-0.102141000	-2.456258000	0.044324000
C	0.647676000	2.901938000	-1.816132000
H	0.206115000	2.048567000	-2.370674000
H	1.673741000	3.079165000	-2.203606000
H	0.040750000	3.808475000	-2.027204000
C	0.938629000	-2.835040000	-1.788130000
H	0.436219000	-3.806213000	-1.985877000
H	1.970729000	-2.894521000	-2.195053000
H	0.392493000	-2.040088000	-2.336616000
C	1.605887000	-3.671415000	0.529648000
H	1.032735000	-4.611966000	0.390409000
H	1.638585000	-3.448492000	1.616489000
H	2.645346000	-3.877546000	0.197182000
C	3.012384000	-1.052754000	-0.018855000
H	3.624007000	-1.967817000	-0.046338000

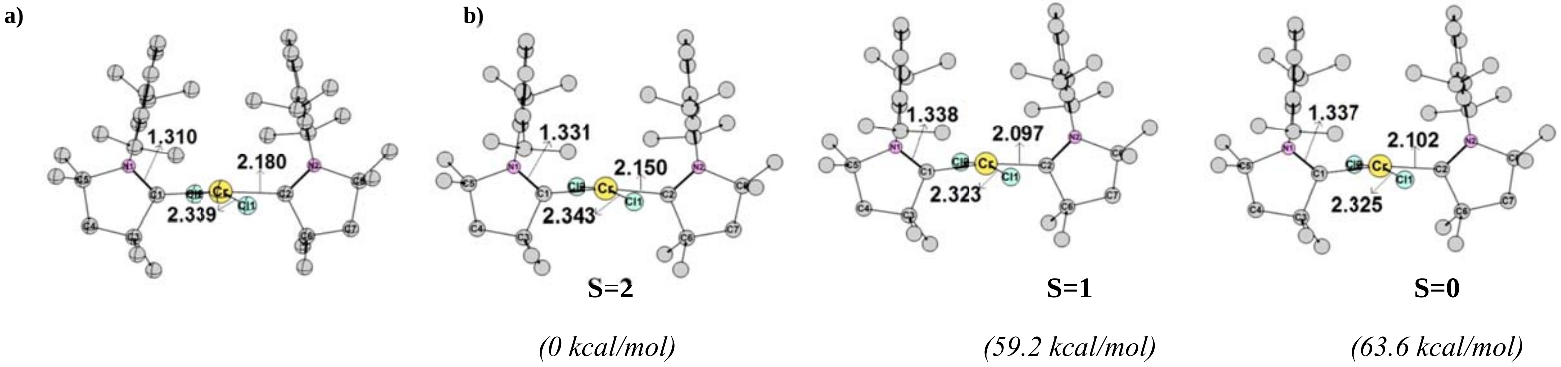

Figure S12: a) Crystal structure of **1** and b) the optimized geometries of **1** in the different spin of states at the BP86/def2-SVP level of theory. The relative energy difference between the different spin states (S, total spin) (at the M06/TZVPP//BP86/def2-SVP level of theory) are given in italics. Hydrogen atoms are omitted for clarity. Distances are given in [Å].

Table S3: Comparison of the selected distances and angles in the crystal structure with optimized (at the BP86/def2-SVP level of theory) structures of **1** in different spin states (S, total spin). Distances are given in [Å] and angles in [°].

Distance/Angle	Crystal structure	S = 2	S = 1	S = 0
Cr–Cl1	2.339	2.343	2.323	2.325
Cr–C12	2.339	2.346	2.326	2.327
Cr–C1	2.180	2.147	2.097	2.098
Cr–C2	2.180	2.150	2.097	2.102
C1–N1	1.310	1.331	1.338	1.337
C2–N2	1.310	1.330	1.339	1.337
C1–C3	1.538	1.541	1.546	1.545
C2–C5	1.538	1.546	1.551	1.551
N1–C4	1.541	1.552	1.548	1.549
N2–C6	1.541	1.549	1.548	1.544
C1–Cr–Cl2	84.7	83.3	83.8	83.8
C1–Cr–Cl1	94.8	94.9	93.8	94.3
Cl2–Cr–Cl1	167.0	170.7	170.2	170.0
Cl2–Cr–C2	94.8	96.0	95.5	95.3
Cl1–Cr–C2	84.7	84.2	85.4	84.9
C1–Cr–C2	175.6	170.2	170.8	170.1
$^{*}\theta_p$(Cr)	1.0	1.6	1.6	1.7

$^{*}\theta_p$ = 360° - (< C1–Cr–Cl2 + < C1–Cr–Cl1 + < Cl2–Cr–C2 + <Cl1-Cr-C2)

a) b)

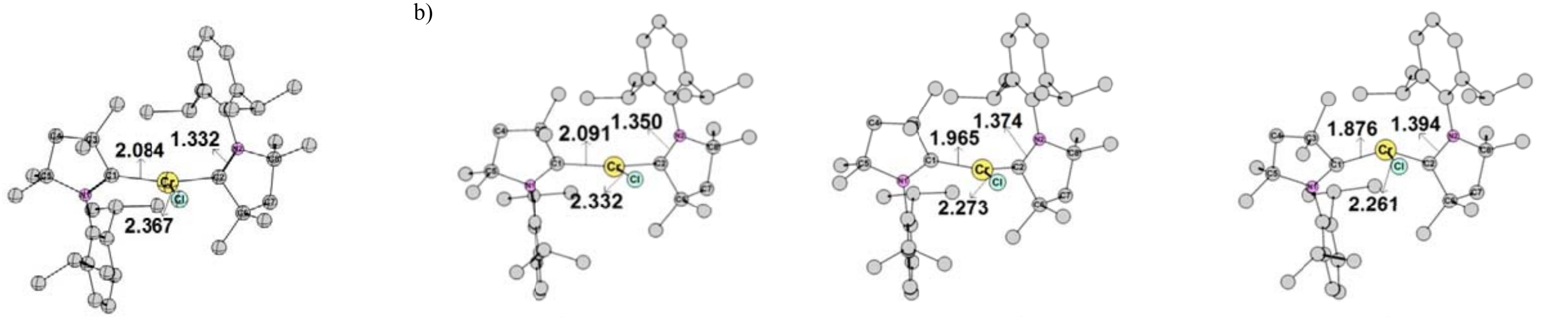

S= 5/2 S= 3/2 S= 1/2

(0 kcal/mol) *(5.8 kcal/mol)* *(38.5 kcal/mol)*

Figure S13: a) Crystal structure of **2** and b) the optimized geometries of **2** in the different spin of states at the BP86/def2-SVP level of theory. The relative energy difference between the different spin states (S, total spin) (at the M06/TZVPP//BP86/def2-SVP level of theory) are given in italics. Hydrogen atoms are omitted for clarity. Distances are given in [Å].

Table S4: Comparison of the selected distances and angles in the crystal structure with optimized (at the BP86/def2-SVP level of theory) structures of **2** in different spin states (S, total spin). Distances are given in [Å] and angles in [°].

**Distance/Angle**	**Crystal structure**	**S=5/2**	**S=3/2**	**S=1/2**
Cr–Cl	2.367	2.332	2.273	2.261
Cr–C1	2.084	2.091	1.965	1.876
Cr–C2	2.093	2.091	1.965	1.910
C1–N1	1.334	1.350	1.374	1.388
C2–N2	1.332	1.350	1.374	1.394
C1–C3	1.535	1.545	1.558	1.577
C2–C6	1.536	1.545	1.558	1.540
C3–C4	1.539	1.553	1.550	1.551
C6–C7	1.537	1.553	1.550	1.664
C4–C5	1.538	1.546	1.544	1.549
C7–C8	1.533	1.546	1.544	1.552
C1–Cr–C2	137.2	147.3	111.4	125.8
C1–Cr–Cl	112.7	106.3	124.3	109.1
C1–Cr–C2	110.7	106.3	124.3	103.9
*θ_p(Cr)	0	0	0	21.3

*θ_p = 360° - (< C1–Cr–C2 + < C1–Cr–Cl + < C1–Cr–C2)

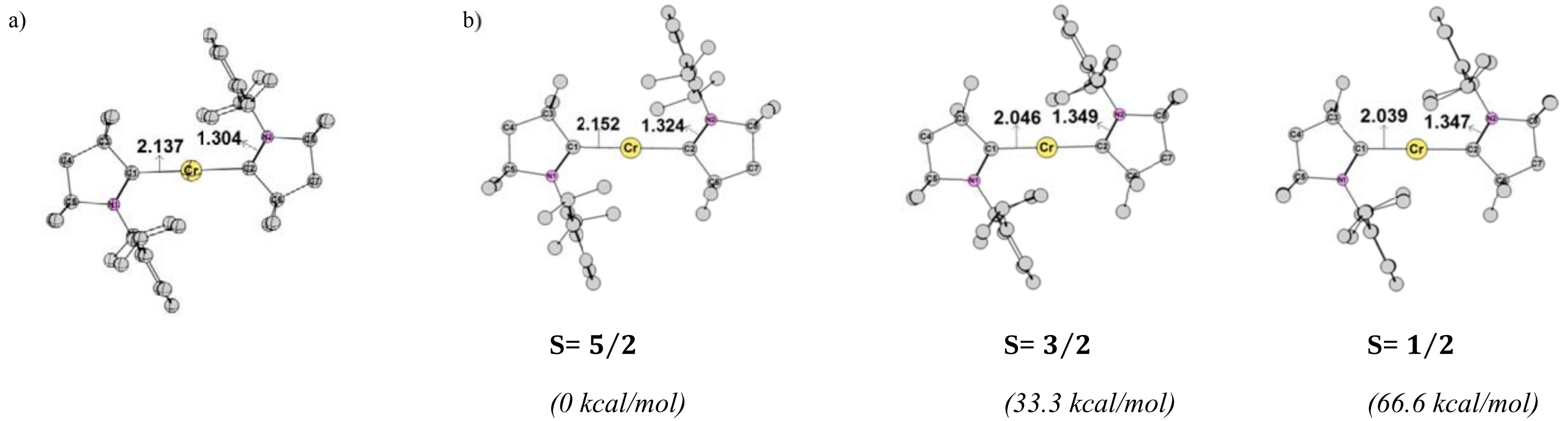

Figure S14: a) Crystal structure of **3** and b) the optimized geometries of **3** in the different spin of states at the BP86/def2-SVP level of theory. The relative energy difference between the different spin states (S, total spin) (at the M06/TZVPP//BP86/def2-SVP level of theory) are given in italics. Hydrogen atoms are omitted for clarity. Distances are given in [Å].

Table S5: Comparison of the selected distances and angles in the crystal structure with optimized (at the BP86/def2-SVP level of theory) structures of **3** in different spin states (S, total spin). Distances are given in [Å] and angles in [°].

Distance/Angle	Crystal structure	S=5/2	S=3/2	S=1/2
Cr–C1	2.137	2.152	2.046	2.039
Cr–C2	2.137	2.152	2.046	2.039
C1–N1	1.304	1.324	1.349	1.347
C2–N2	1.304	1.324	1.349	1.347
C1–C3	1.510	1.530	1.539	1.540
C2–C6	1.510	1.530	1.539	1.540
C3–C4	1.517	1.558	1.559	1.559
C6–C7	1.517	1.558	1.559	1.559
C4–C5	1.503	1.548	1.548	1.548
C7–C8	1.503	1.548	1.548	1.548
C1–Cr–C2	180.0	180.0	180.0	180.0
N1–C1–C3	108.7	108.8	107.9	107.8
N1–C1–Cr	122.3	124.4	120.7	120.7
C3–C1–Cr	129.0	126.9	131.2	131.3

Table S6: Schematic representation, natural charge and bond order by natural population analysis and the dissociation energy (D_e) (in kcal/mol) for one Cr–C_{cAAC} bond of $(cAAC)_2CrCl_2$ (**1**) in the different spin states (S = 0, S = 1 and S = 2, where S is the total spin) at the M06/def2-TZVPP//BP86/def2-TZVPP level of theory.

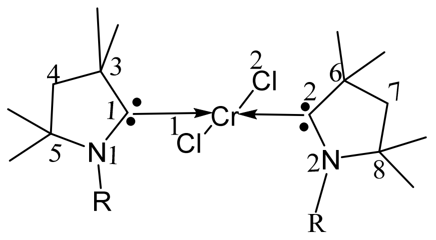

1

**Atom/Group**	**Charge**		
	S = 0	S = 1	S = 2
Cr	0.50	0.70	0.55
C1	0.17	0.09	0.16
C2	0.17	0.07	0.17
N1	-0.14	-0.13	-0.14
N2	-0.45	-0.46	-0.45
cAAC	0.21	0.14	0.23
Cl1	-0.46	-0.50	-0.50
Cl2	-0.46	-0.49	-0.50
**Bond**	**Bond Order**		
	S = 0	S = 1	S = 2
Cr–C1	0.49	0.57	0.42
Cr–C2	0.49	0.61	0.42
C1–N1	1.45	1.40	1.47
C2–N2	1.45	1.39	1.47
**Dissociation energy ($D_{e)}$ for Cr–C_{cAAC} bond**			
	S = 0	S = 1	S = 2
	18.3	21.5	51.1

Table S7: Schematic representation, natural charge and bond order by natural population analysis and the dissociation energy (D_e) (in kcal/mol) for one Cr–C_{cAAC} bond of $(cAAC)_2CrCl$ (**2**) in the different spin states (S = 1/2, S = 3/2 and S = 5/2, where S is the total spin) at the M06/def2-TZVPP//BP86/def2-TZVPP level of theory.

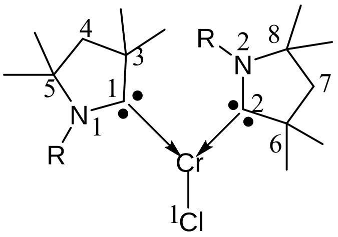

2

**Atom/Group**	**Charge**		
	S = 1/2	S = 3/2	S = 5/2
Cr	0.67	0.60	0.54
C1	-0.09	-0.04	-0.03
C2	-0.05	-0.04	-0.03
N1	-0.49	-0.47	-0.45
N2	-0.48	-0.47	-0.45
cAAC	-0.12	-0.06	0.01
Cl	-0.43	-0.47	-0.56

**Bond**	**Bond Order**		
	S = 1/2	S = 3/2	S = 5/2
Cr–C1	1.03	0.80	0.49
Cr–C2	0.95	0.80	0.49
C1–N1	1.15	1.25	1.37
C2–N2	1.16	1.25	1.37

**Dissociation energy (D_e) for Cr–C_{cAAC} bond**			
	S = 1/2	S = 3/2	S = 5/2
	29.4	45.7	48.6

Table S8: Schematic representation, natural charge and bond order by natural population analysis and the dissociation energy (D_e) (in kcal/mol) for one Cr–C_{cAAC} bond of $(cAAC)_2Cr^+$ (**3**) in the different spin states (S = 1/2, S = 3/2 and S = 5/2, where S is the total spin) at the M06/def2-TZVPP//BP86/def2-TZVPP level of theory.

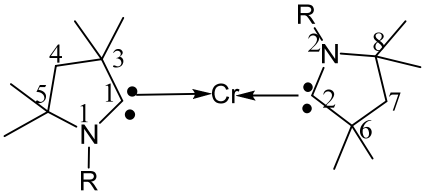

3

**Atom/Group**	**Charge**		
	S = 1/2	S = 3/2	S = 5/2
Cr	0.76	0.90	0.55
C1	-0.07	-0.14	0.01
C2	-0.07	-0.14	0.01
N1	-0.45	-0.45	-0.41
N2	-0.45	-0.45	-0.41
cAAC	0.12	0.05	0.23

**Bond**	**Bond Order**		
	S = 1/2	S = 3/2	S = 5/2
Cr–C1	0.58	0.56	0.32
Cr–C2	0.58	0.56	0.32
C1–N1	1.38	1.34	1.50
C2–N2	1.38	1.34	1.50

**Dissociation energy (D_e) for Cr–C_{cAAC} bond**			
	S = 1/2	S = 3/2	S = 5/2
	46.4	63.0	79.7

Table S9: Net NBO spin density on selected atoms in **1** in quintet, **2** in sextet and **3** in sextet spin state by the natural population analysis at the M06/def2-TZVPP//BP86/def2-TZVPP level of theory.

	NBO spin density		
	**1**	**2**	**3**
Cr	3.85	4.25	4.65
C1	-0.04	0.14	0.01
C2	-0.04	0.14	0.01
N1	0.03	0.01	0.06
N2	0.03	0.01	0.06
Cl1	0.02	0.04	-
Cl2	0.01	-	-

References:

(1) (a) Lavallo, V.; Canac, Y.; Präsang, C.; Donnadieu, B.; Bertrand, G. *Angew. Chem. Int. Ed.* **2005**, *44*, 5705–5709; (b) Jazzar, R.; Dewhurst, R. D.; Bourg, J.-B.; Donnadieu, B.; Canac, Y.; Bertrand, G. *Angew.Chem. Int. Ed.* **2007**, *46*, 2899–2902.

(2) (a) Stalke, D. *Chem. Soc. Rev.* **1998**, *27*, 171-178. (b) Kottke, T.; Stalke, D. *J. Appl. Crystallogr.* **1993**, *26*, 615-619.

(3) Schulz, T.; Meindl, K.; Leusser, D.; Stern, D.; Graf, J.; Michaelsen, C.; Ruf, M.; Sheldrick, G. M.; Stalke, D. *J. Appl. Crystallogr.* **2009**, *42*, 885-891.

(4) SAINT, Bruker AXS Inc., Madison, Wisconsin (USA) 2000.

(5) Sheldrick, G. M. *SADABS*, Universität Göttingen, Germany, 2000.

(6) (a) Sheldrick, G. M. *Acta Crystallogr.*, Sect. A **1990**, *46*, 467−473. (b) Sheldrick, G. M. *Acta Crystallogr.*, Sect. A **2008**, *64*, 112−122. (c) Müller, P.; Herbst-Irmer, R.; Spek, A. L.; Schneider, T. R.; Sawaya, M. R. In *Crystal Structure Refinement−A Crystallographer's Guide to SHELXL, IUCr Texts on Crystallography*; P. Müller, Ed.; Oxford University Press: Oxford, U.K., 2006; Vol. 8. (d) Hübschle, C. B.; Sheldrick, G. M.; Dittrich, B. *J. Appl. Crystallogr.* **2011**, *44*, 1281−1284.

(7) Jones, C.; Dange, D.; Stasch, A. *J. Chem Crystallogr.* **2012**, *42*, 494–497.

(8) Haberditzel, W. *Angew. Chem. Int. Ed. Engl.* **1966**, *5*, 288. (b) Bain, G. A.; Berry, J. F. *J. Chem. Educ.* **2008**, *85*, 532.

(9) (a) Krzystek, J.; Ozarowski, A.; Telser, *Coordin. Chem. Rev.* **2006,** *250* (17–18), 2308-2324; (b) Krzystek, J.; Zvyagin, S. A.; Ozarowski, A.; Trofimenko, S.; Telser, J. *J. Magn. Reson.* **2006,** *178* (2), 174-183; (c) Liu, W.; Christian, J. H.; Al-Oweini, R.; Bassil, B. S.; van Tol, J.; Atanasov, M.; Neese, F.; Dalal, N. S.; Kortz, U. *Inorg. Chem.* **2014,** *53* (17), 9274-9283.

(10)(a) Becke, A. D. *Phys. Rev. A* **1988**, *38*, 3098-3100; (b) Perdew, J. P. *Phys. Rev. B* **1986**, *33*, 8822-8824.

(11)(a) Frisch, M. J.; Trucks, G. W.; Schlegel, H. B.; Scuseria, G. E.; Robb, M. A.; Cheeseman, J. R.; Scalmani, G.; Barone, V.; Mennucci, B.; Petersson, G. A.; Nakatsuji, H.; Caricato, M.; Li, X.; Hratchian, H. P.; Izmaylov, A. F.; Bloino, J.; Zheng, G.; Sonnenberg, J. L.; Hada, M.; Ehara, M.; Toyota, K.; Fukuda, R.; Hasegawa, J.; Ishida, M.; Nakajima, T.; Honda, Y.; Kitao, O.; Nakai, H.; Vreven, T.; Montgomery, J. A. Jr.; Peralta, J. E.; Ogliaro, F.; Bearpark, M.; Heyd, J. J.; Brothers, E.; Kudin, K. N.; Staroverov, V. N.; Keith, T.; Kobayashi, R.; Normand, J.; Raghavachari, K.; Rendell, A.; Burant, J. C.; Iyengar, S. S.; Tomasi, J.; Cossi, M.; Rega, N.; Millam, J. M.; Klene, M.; Knox, J. E.; Cross, J. B.; Bakken, V.; Adamo, C.; Jaramillo, J.; Gomperts, R.; Stratmann, R. E.; Yazyev, O.; Austin, A. J.; Cammi, R.; Pomelli, C.; Ochterski, J. W.; Martin, R. L.; Morokuma, K.; Zakrzewski, V. G.; Voth, G. A.; Salvador, P.; Dannenberg, J. J.; Dapprich, S.; Daniels, A. D.; Farkas, O.; Foresman, J. B.; Ortiz, J. V.; Cioslowski, J.; Fox, D. J.; Gaussian, Inc., Wallingford CT, **2010**.

(12) (a) Andrae, D.; Haeussermann, U.; Dolg, M.; Stoll, H.; Preuss, H.; *Theor. Chim. Acta*, **1990**, *77*, 123-141. (b) Metz, B.; Stoll, H.; Dolg, M.; *J. Chem. Phys.*, **2000**, *113*, 2563-2569. (c) Peterson, K. A.; Figgen, D.; Goll, E.; Stoll, H.; Dolg, M.; *J. Chem. Phys.*, **2003**, *119*, 11113-11123. (d) Leininger, T.; Nicklass, A.; Kuechle, W.; Stoll, H.; Dolg, M.; Bergner, A.; *Chem. Phys. Lett.*, **1996**, *255*, 274-280. (e) Kaupp, M.; Schleyer, P. V.; Stoll, H.; Preuss, H.; *J. Chem. Phys.*, **1991**, *94*, 1360-1366.

(13) Zhao, Y.; Truhlar, D. G. *Theor. Chem. Acc.* **2008**, *120*, 215–241.

(14) (a) Reed, A. E.; Curtiss, L. A.; Weinhold, F. *Chem. Rev.* **1988**, *88*, 899–926. (b) NBO 6.0. Glendening, E. D.; Badenhoop, J. K.; Reed, A. E.; Carpenter, J. E.; Bohmann, J. A.; Morales, C. M.; Landis, C. R.; Weinhold F. (Theoretical Chemistry Institute, University of Wisconsin, Madison, WI, 2013); http://nbo6.chem.wisc.edu/.
